# Genome‐Wide and Multi‐Omics Analysis Identified DNA‐Interacting Circular RNA circFOXK2 as MLL1 Regulator for Histone H3K4 Methylation in Pancreatic Ductal Adenocarcinoma

**DOI:** 10.1002/advs.77107

**Published:** 2026-10-29

**Authors:** Chi Hin Wong, Jessica Jazmin Peña‐Paladines, Sum Yin Valerie Leung, Ka‐Fai To, Kenneth Siu Ho Chok, Yangchao Chen

**Affiliations:** ^1^ School of Biomedical Sciences Faculty of Medicine The Chinese University of Hong Kong Shatin New Territories Hong Kong; ^2^ Shenzhen Research Institute The Chinese University of Hong Kong Shenzhen China; ^3^ Centre for Immuno‐Oncology Nuffield Department of Medicine University of Oxford Oxford UK; ^4^ Department of Anatomical and Cellular Pathology Prince of Wales Hospital The Chinese University of Hong Kong Shatin Hong Kong; ^5^ Department of Surgery Faculty of Medicine The Chinese University of Hong Kong Shatin Hong Kong

**Keywords:** circular RNA, H3K4 methylation, MLL1, pancreatic ductal adenocarcinoma, PPIE

## Abstract

Pancreatic ductal adenocarcinoma (PDAC) is one of the most devastating malignancies with high lethality despite the current therapeutic interventions. Circular RNAs (circRNAs) play important roles in many biological processes and disease development. However, the underlying mechanism remains largely unexplored. To identify novel druggable targets, we aimed to elucidate the mechanisms contributing to the critical roles of circRNAs in PDAC. We performed a genome‐wide and multi‐omics investigation on the circFOXK2‐DNA interaction in PDAC progression. Single‐cell ATAC‐sequencing analysis using the publicly available dataset revealed the importance of the circFOXK2‐DNA interaction network in intratumoral heterogeneity. Through circRNA‐DNA interaction by complementary base pairing, circFOXK2 recognizes targets at different genomic locations: enhancer, promoter, and intragenic region. CircFOXK2 forms a docking pocket by intramolecular base pairing for the N‐terminal RNA‐recognition motif of PPIE, disrupting the PPIE‐MLL1 interaction. This removes the PPIE inhibitory effects on MLL1 for gene‐activating H3K4 methylation. Specifically, circFOXK2 promotes TMSB15B and BCAS1 expressions by enhancing H3K4me3 at the promoter. At the gene body, circFOXK2 facilitates transcription elongation of TRIO and LAMA4 by intragenic H3K4me3. At the distal intergenic enhancer, circFOXK2 promotes H3K4me1 of CYP26A1 and TCP11 for gene activation. These results highlight the importance and therapeutic potential of circRNA‐DNA interaction in gene regulation.

## Graphical abstract

Circular RNA circFOXK2 is frequently upregulated in pancreatic ductal adenocarcinoma to promote tumor heterogeneity, growth, and metastasis. Through circRNA‐DNA interaction at different genomic locations, circFOXK2 removes the PPIE inhibitory effects on MLL1 for gene‐activating H3K4 methylation of the oncogenes. This highlights the importance of circRNAs in gene regulation through circRNA‐DNA interaction and uncovered the novel therapeutic direction targeting circFOXK2‐DNA interaction.

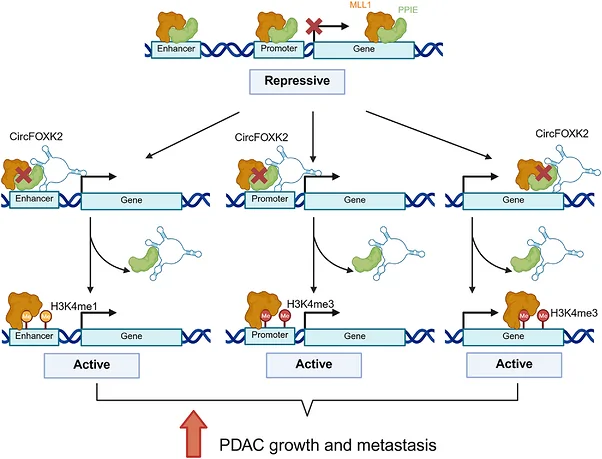

**Abbreviations**
BCAS1: brain enriched myelin associated protein 1
circRNA: Circular RNA
CYP26A1: cytochrome P450 26A1
H3K4me1: histone H3 lysine 4 monomethylation
H3K4me3: histone H3 lysine 4 trimethylation
LAMA4: laminin subunit alpha 4
miRNAs: microRNAs
MLL1: mixed lineage leukemia 1
PDAC: pancreatic ductal adenocarcinoma
PPIE: peptidylprolyl isomerase E
RNA PolII Ser2: RNA Polymerase II serine 2 phosphorylation
RNA PolII: RNA Polymerase II
RRM: RNA‐recognition motif
TCP11: t‐complex 11
TMSB15B: thymosin beta 15B
TRIO: trio rho guanine nucleotide exchange factor

## 1 Introduction

Circular RNAs (circRNAs) are a distinct group in the non‐coding RNA family, featuring a covalently closed circular structure. Although most lack protein‐coding ability, circRNAs still play critical roles in many biological processes and disease development through regulating gene expression and protein activity. Regarding pancreatic ductal adenocarcinoma (PDAC), the fourth leading cause of cancer‐related death, with the overall survival rate of 13% [1], dysregulated circRNAs were frequently found to regulate tumor growth, metastasis, and drug resistance [2]. However, the detailed functional roles and regulatory mechanisms of circRNAs are still largely unexplored. Due to the lack of effective therapeutics and extremely high lethality, it is urgently needed to explore novel mechanisms of circRNAs for identifying novel druggable targets in PDAC.

CircRNAs frequently exert their functional and mechanistic roles in cancer progression through molecular interactions. Functioning as a microRNA (miRNA) sponge is the first reported role of circRNAs [3]. Through binding to the miRNAs by complementary base‐pairing, circRNAs can inhibit target miRNAs to modulate downstream mRNAs' expression. Notably, many circRNAs are expressed at a much lower abundance than miRNAs and harbor only a few miRNA‐binding sites [4, 5], suggesting that miRNA sponging may not be the major circRNA‐regulatory mechanism. Meanwhile, emerging studies identified additional roles of circRNAs through protein interaction and RNA‐binding. CircRNAs stabilize their interacting proteins by blocking ubiquitination or, conversely, promote protein degradation by recruiting ligase [2]. Also, circRNAs compete with their cognate mRNA for protein binding to inhibit translation [2]. The discovery of novel molecular mechanisms suggests the critical roles of circRNAs in PDAC are not yet fully identified.

In our previous study, we demonstrated that the upregulated circFOXK2 in PDAC tumors promoted tumor growth and liver metastasis [6]. In the current study, we further explored the detailed molecular mechanisms contributing to its oncogenic roles in PDAC. Genome‐wide investigation using chromatin isolation by RNA purification (ChIRP)‐sequencing and transcriptome profiling identified novel mechanistic roles of circFOXK2 through circRNA‐DNA interaction. Single‐cell investigation uncovered the circFOXK2‐DNA network in PDAC intratumoral heterogeneity. Furthermore, multi‐omics, functional, and clinical investigations were conducted to elucidate the novel mechanistic roles of circFOXK2‐DNA interaction in regulating epigenetics and gene expression. Through circRNA‐DNA interaction by complementary base pairing, circFOXK2 recognized downstream targets at different genomic locations: enhancers, promoters, and intragenic regions. At the target genes, circFOXK2 formed a docking pocket by intramolecular base pairing for the N‐terminal RNA‐recognition motif of peptidylprolyl isomerase E (PPIE), disrupting the PPIE‐mixed lineage leukemia 1 (MLL1) interaction. This removed the PPIE inhibitory effects on MLL1 for gene‐activating histone H3 lysine 4 methylation (H3K4me). These implied a novel oncogenic mechanism of circFOXK2 in cancer progression and the therapeutic potential of targeting circRNA‐DNA interaction in PDAC.

## 2 Materials and Methods

### 2.1 Cell Lines and Cell Culture

PDAC cell lines PANC‐1, CFPAC‐1, SW1990, CAPAN‐2, and BxPC‐3 were obtained from the American Type Culture Collection (Manassas, VA, USA). Human pancreatic ductal epithelial (HPDE) cell line was generously provided by Dr. Tsao (University Health Network, Ontario Cancer Institute, and Princess Margaret Hospital Site, Toronto) [7]. All cell lines were verified by short tandem repeat profiling at GENEWIZ, Inc. within 6 months of use, and were cultured under the conditions as described previously [8].

### 2.2 PDAC Clinical Samples

84 pairs of PDAC primary tumor and adjacent non‐tumor tissues were obtained from patients who underwent pancreatic resection at the Prince of Wales Hospital, Hong Kong. The study was carried out with the approval of the Joint CUHK‐NTEC Clinical Research Ethics Committee (Ref. No.: 2020.042). Written informed consent forms were signed by the patients. The clinical information of the patients is listed in Table 1. All specimens were fixed and embedded in paraffin.

**TABLE 1:** Correlations between circFOXK2 and clinicopathologic features in PDAC.

Clinicopathological characteristics	Downregulated/ Unchanged circFOXK2	Upregulated circFOXK2	*p*‐value
Gender			
Female	22 (64.7%)	12 (24%)	**0.0002**
Male	12 (35.3%)	38 (76%)	
Age, years			
< 60	13 (41.2%)	12 (24%)	0.161
≥ 60	21 (58.8%)	38 (76%)	
Tumor size, cm			
≤ 2.5	14 (41.2%)	20 (40%)	0.914
> 2.5	20 (58.8%)	30 (60%)	
Histological grade			
Poorly differentiated	3 (10.7%)	0 (0%)	**0.006**
Moderately differentiated	25 (89.3%)	41 (87.2%)	
Well differentiated	0 (0%)	5 (12.8%)	
Tumor stage			
I	10 (41.7%)	16 (32.7%)	0.813
II‐IV	24 (58.3%)	33 (67.3%)	

### 2.3 Plasmid and Oligonucleotide Transfection

CircFOXK2 overexpression plasmid was constructed as previously described [6]. PPIE expression plasmid was constructed using Champion pET SUMO Expression System (Thermofisher Scientific, Waltham, MA, USA). Mutation of the plasmids was achieved using KAPA HiFi DNA Polymerase (KapaBiosystem, St. Louis, MO, USA) and primers with the mutation site. SiRNAs were purchased from IGE Biotechnology (China). The sequences were presented in Table S1. Plasmids and siRNA transfection were performed by Lipofectamine 3000 (Thermofisher Scientific), according to the manufacturer's protocol.

### 2.4 RNA‐Sequencing

Paired‐end (PE150, sequencing reads 150bp) RNA sequencing was performed at RiboBio (China) using the Illumina Hiseq 3000 platform, and PE100 RNA sequencing was performed at BGI (China) using the DNBseq G400 platform. After removing adaptors, low‐quality reads, and rRNA reads, clean reads were mapped to the human reference genome (hg38) by HISAT2. Differentially expressed genes with a fold change > 2 and an adjusted *p*‐value of < 0.05 were identified by edgeR.

### 2.5 Chromatin Isolation by RNA Purification (ChIRP)‐Sequencing

ChIRP in PANC‐1 cells was performed as previously described [9]. Briefly, cells were fixed with 4% paraformaldehyde. Then the crosslinked chromatin was sonicated and hybridized with five biotin‐labeled oligos antisense to the specific back‐splicing junction of circFOXK2 at 37°C for 4 h with rotation. The hybridized circFOXK2‐chromatin was captured by streptavidin‐labeled beads, followed by DNA purification. Then, the isolated DNA was subjected to sequencing at BGI (China) using the DNBSEQ platform. The clean reads were mapped to the human reference genome (hg38) by Bowtie2. The peaks of circFOXK2‐interacting DNA were identified by MACS2.

### 2.6 Quantitative Reverse Transcription PCR (qRT‐PCR)

Total RNA was extracted by TRIzol Reagent (Thermofisher Scientific). cDNA was synthesized using PrimeScript RT Kit (Takara, Japan) according to the manufacturer's instructions. Quantitative PCR was performed by ABI QuantStudio 7 (Flex) Real‐time PCR System (Thermofisher Scientific) using Power Up ^TM^ SYBR Green PCR Master Mix (Thermofisher Scientific). Gene expression was normalized with the housekeeping gene GAPDH. The PCR primer sequences are listed in Table S2.

### 2.7 Protein Expression and Purification

PPIE expression construct was transformed into *Escherichia coli* BL‐21(DE3) cells. Protein expression was induced by 1 mm IPTG, followed by overnight incubation at 30°C. Cell pellet was collected by centrifugation and lysed in lysis buffer (3 mm KH_2_PO_4_, 47 mm K_2_HPO_4_, 100 mm KCl, 400 mm NaCl, 10% glycerol, 0.5% Triton X‐100, 10 mm imidazole, pH 7.8), and then by sonication at 4°C. The lysate was cleared by centrifugation, followed by loading the supernatant onto a HisTrap‐HF column (GE Healthcare) pre‐equilibrated with NPI‐20 wash buffer (50 mm NaH_2_PO_4_, 300 mm NaCl, 20 mm imidazole, pH 8.0). Then, the column was washed with NPI‐20 wash buffer and was eluted with NPI‐500 elution buffer (50 mm NaH_2_PO_4_, 300 mm NaCl, 500 mm imidazole, pH 8.0).

### 2.8 Immunoblotting

The whole cell extract was prepared by lysing cells in RIPA lysis buffer with proteinase and phosphatase inhibitors (MedChemExpress, Monmouth Junction, NJ, USA). Proteins were resolved by SDS‐PAGE at different percentages, transferred to PVDF membrane and immunoblotted overnight at 4°C with antibodies against PPIE (rabbit; ab154865 Abcam, Cambridge, UK; 1:1000); MLL1 (rabbit; LS‐C287274 LSBio, Newark, CA, USA; 1:1000); histone H3K4me1 (rabbit; 07–436 Millipore, Burlington, MA, USA; 1:1000), histone H3K4me3 (rabbit; CS200580 Millipore; 1:1000); histone H3 (rabbit; 4620 Cell Signaling Technology, Danvers, MA, USA; 1:1000); TRIO (rabbit; DF2685 Affinity Biosciences, Cincinnati, OH, USA; 1:1000); LAMA4 (rabbit; DF3617 Affinity Biosciences; 1:1000); TMSB15B (rabbit; HPA064607 Sigma–Aldrich; 1000); BCAS1 (rabbit; 25776‐1‐AP Proteintech, Rosemont, IL, USA; 1:1000); CYP26A1 (rabbit; A5982 Abclonal, Woburn, MA, USA; 1:1000); TCP11 (rabbit; 14606‐1‐AP Proteintech; 1:1000); and GAPDH (mouse; AC002 Abclonal; 1:1000). Chemiluminescent signals were developed using Immobilon Western Chemiluminescent HRP Substrate (Millipore).

### 2.9 Immunohistochemistry (IHC)

Immunohistochemical staining was performed using Mouse and Rabbit Specific HRP/DAB IHC Detection Kit (abcam). The PDAC FFPE sections were probed with antibodies against H3K4me3 (rabbit; CS200580 Millipore; 1:100); H3 (rabbit; 4620 Cell Signaling Technology; 1:100); TRIO (rabbit; DF2685 Affinity Biosciences; 1:100); LAMA4 (rabbit; DF3617 Affinity Biosciences; 1:100); TMSB15B (rabbit; HPA064607 Sigma–Aldrich; 100); BCAS1 (rabbit; 25776‐1‐AP Proteintech; 1:100); CYP26A1 (rabbit; A5982 Abclonal; 1:100); and TCP11 (rabbit; 14606‐1‐AP Proteintech; 1:100), were counter‐stained with hematoxylin, and were mounted. A scoring system, based on the percentage of positive cells and staining intensity under the microscope with 100X magnification, was used to quantify the staining. 4 categories (0, 1, 2, and 3) were demoted as 0%, 1–10%, 10–50, and > 50%, respectively.

### 2.10 Immunofluorescence

The PDAC FFPE sections were probed with Alexa Fluor 647‐labeled anti‐MLL1 antibodies (rabbit; 1:100) and Alexa Fluor 488‐labeled anti‐PPIE antibodies (rabbit; ab154865 Abcam; 1:100) at 4°C, counter‐stained with DAPI, and mounted. The fluorescent signals were observed under Nikon Ti2‐E Inverted Fluorescence Microscope. To quantify MLL1 and PPIE co‐localization, fluorescence images were analyzed using ImageJ with the JACoP plug‐in, and Mander's overlap coefficient was calculated for quantification [10].

### 2.11 RNA Immunoprecipitation

RNA immunoprecipitation was performed using Magna RIP RNA Binding Protein Immunoprecipitation Kit (Millipore) according to the manufacturer's instructions. Briefly, cells were washed twice with ice‐cold PBS, followed by cell lysis using RIP lysis buffer. Magnetic beads were washed twice with RIP wash buffer, followed by incubation with 2 µg antibody against PPIE (rabbit; ab154865 Abcam) or negative control IgG for 30 min at room temperature. Immunoprecipitation was performed by incubating cell lysate with the magnetic bead–antibody complex at 4°C overnight. Then, the beads were washed six times with RIP wash buffer, followed by proteinase K digestion at 37°C for 30 min. RNA was purified by phenol:chloroform:isoamylalcohol (Invitrogen). qRT‐PCR was performed to analyze the enrichment of RNAs with target proteins (relative to the negative control).

### 2.12 Electrophoretic Mobility Shift Assay (EMSA)

In vitro transcribed circFOXK2 was incubated with purified human PPIE protein in binding buffer with 4% glycerol for 30 min at room temperature. Then, circFOXK2‐PPIE complex was separated on a 5% polyacrylamide gel, followed by nucleic acid staining by Gel‐Red (Beyotime, China).

### 2.13 Modeling of the circFOXK2‐PPIE Interaction

The three‐dimensional (3D) structure of circFOXK2 was modeled by the computational methods 3dRNA [11]. 3D structure of PPIE was obtained from the Protein Data Bank [12]. Then, RBPmap [13] and HNADOCK [14] with the default settings were used to model the circFOXK2‐PPIE interaction.

### 2.14 Co‐Immunoprecipitation (Co‐IP)

Co‐IP was performed using Pierce Classic Magnetic IP/Co‐IP Kit (Thermofisher Scientific) according to the manufacturer's protocol. Briefly, the cells were treated with lysis buffer, followed by incubation with antibodies against MLL1 antibody (Millipore, ABE240) at 4°C overnight with rotation. Protein A/G magnetic beads were added to the samples to collect the immunoprecipitated protein complex. The eluted protein complex was subjected to immunoblotting using an antibody against PPIE (rabbit; ab154865 Abcam).

### 2.15 Chromatin Immunoprecipitation (ChIP)

ChIP was performed using EZ‐Magna ChIP HiSens kit (Millipore) according to the manufacturer's protocol. Briefly, chromatins were cross‐linked with 1% formaldehyde, isolated, and sonicated into fragments of 200–600 bp. Cross‐linked chromatin was incubated at 4°C overnight with IgG (negative control), anti‐RNA Polymerase II antibody (Millipore, 05–623), anti‐RNA Polymerase II Phosphorylated Ser2 antibody (Covance, Princeton, NJ, USA, MMS‐129R), anti‐MLL1 antibody (Millipore, ABE240), anti‐H3K4me1 antibody (Millipore, 07–436), and anti‐H3K4me3 antibody (Millipore, CS200580). qRT‐PCR was performed to analyze the enrichment of DNAs with target proteins.

### 2.16 Nuclear Accessibility Assay

The chromatin accessibility of the genes was assayed with DNase I digestion, which mimicked the binding of RNA Polymerase II, according to our previous study [15]. Briefly, 1 × 10^7^ cells were washed with cold PBS twice, followed by nucleus isolation by NP‐40 nuclei isolation buffer. The isolated nucleus was digested in DNase I digestion buffer (6 mm CaCl_2_, 88.5 mm NaCl, 54 mm KCl, 0.9 mm EDTA, 0.45 mm EGTA, 0.45 mm spermidine, and 13.5 mm Tris, pH 8) with 25 U DNase I (Qiagen) at 37°C for 3 min [16]. DNA was extracted by phenol: chloroform: isoamylalcohol, followed by ethanol precipitation. The chromatin accessibility was analyzed by qPCR.

### 2.17 Nuclear Run‐On Assay

Nuclear run‐on assay was performed as previously described [15]. Nuclei were isolated using the nuclei isolation buffer. Nuclear Run‐on assay was performed by incubating the nuclei at 37°C for 30 min in transcription buffer (7% glycerol 5 mm MgCl_2_, 75 mm KCl, 50 µm EDTA, 25 mm Tris and 25 mm HEPES, pH 7.5) containing nucleoside triphosphates (0.35 mm ATP, GTP and CTP, 0.4 µm UTP and 0.25 mm biotin‐labeled UTP) (Biosearch Technologies, the United Kingdom), followed by RNA isolation by TRIZOL reagent. Nascent transcribed RNA was isolated by Dynabeads MyOne Streptavidin C1 magnetic beads (Thermofisher Scientific), followed by qRT‐PCR analysis.

### 2.18 Cell Viability Assay

3‐(4,5‐dimethylthiazol‐2‐yl)‐5‐(3‐carboxymethoxyphenyl)‐2‐(4‐sulfophenyl)‐2H‐tetrazolium (MTS) assay was performed to examine the treatment effect on cell viability. 2500 cells were seeded in a 96‐well cell culture plate. After 24, 72, and 120 h, 0.33 mg/mL MTS reagent was added to the cells. After 2 h, absorbance at 495 nm was measured using a microplate spectrophotometer (Thermofisher Scientific).

### 2.19 Anchorage‐Dependent Colony Formation Assay

For the colony formation assay, 1 × 10^3^ cells were seeded in 6‐well plates and incubated at 37°C for 2–3 weeks [17]. Then, the cells were fixed with 3.7% formaldehyde, permeabilized by methanol, and stained by 0.5% crystal violet. The stained colonies were counted.

### 2.20 Migration Assay

Wound healing cell migration assay was performed by seeding the cells in a migration insert [18]. When the cells attached to the culture plate, the insert was removed, and the cells were maintained in serum‐free cell culture medium. The wound was imaged under light microscopy at 0, 24, and 48 h, and the wound width was measured.

### 2.21 Invasion Assay

The Trans‐well cell invasion assay was performed as described previously. The upper chamber of the trans‐well insert was coated with Matrigel (Corning, Glendale, AZ, USA). Then, 25 000 cells were seeded in the insert in a serum‐free culture medium. Then the insert with seeded cells was placed in a plate well with a complete culture medium. After 24 to 72 h, the trans‐well insert was fixed with 3.7% formaldehyde, permeabilized by methanol, and stained with 0.5% crystal violet. The non‐invaded cells were removed, and the invaded cells were counted.

### 2.22 Analysis of Publicly Available Datasets

Transcript expression comparison between PDAC and non‐tumor tissues, based on publicly available TCGA and GTEx data, was performed using GEPIA2 [19]. Publicly available ChIP sequencing (ChIP‐seq), DNase‐sequencing (DNase‐seq), and single‐cell Assay for Transposase Accessible Chromatin sequencing (scATAC‐seq) datasets were obtained from the ENCODE portal with the following identifiers: ENCSR713YDD, ENCSR211SBE, ENCSR912QMS, ENCSR000EXI, ENCSR000DXB, ENCSR236KPK, ENCSR999VDH, ENCSR589HAC, ENCSR000EPT, ENCSR974TUB, ENCSR000FAI, ENCSR022UWN, ENCSR737GTV, and ENCSR000EEJ [20]. Enhancer information of the target genes was obtained from the USCS genome browser.

For ChIP‐seq and DNase‐seq data analysis, after the initial control and removal of sequencing adaptors, the sequencing reads were mapped to the human reference genome hg38 with bowtie2 (version 2.3.4.3) [21]. The enriched binding sites in ChIP‐seq experiments or the open chromatin regions in DNase‐seq experiments were identified by peak calling with MACS2 (Version 2.2.9.1) (False discovery rate < 0.05) [22].

For scATAC‐seq data analysis, barcodes were attached to the fastq files using Sinto (version 0.9.0) [23]. After initial quality control, the sequencing reads were mapped to the human reference genome hg38 with BWA (version 0.7.17) [24]. Open chromatin regions were identified by peak calling with MACS2 (Version 2.2.9.1) (False discovery rate < 0.05) [22]. Then, a scATAC count matrix with cell barcodes and peaks information was built using epiScanpy (Version 0.3.2) [25]. Features covered in too few cells and those not containing enough features were removed. The first 50 Principal Component Analysis (PCA) components were projected on the two‐dimensional Uniform Manifold Approximation and Projection (UMAP) representation. Cells were clustered by Leiden community detection algorithm at the resolution of 0.5. Differential active chromatin analysis between clusters was performed using “tl.rank_features” function, which outputs the significant features in each cluster. Gene activities of each cluster were evaluated for Kyoto Encyclopedia of Genes and Genomes (KEGG) pathway and Gene Ontology (GO) analysis. For determining the metastatic ability, we examined the gene activities of the related gene set from Zhang et al. [26].

### 2.23 Statistical Analysis

GraphPad Prism 9 (GraphPad Software) was used for statistical analysis. A two‐tailed Student's *t*‐test was used to compare the differences between two groups, unless noted otherwise. Data is represented as mean ± SD. A *p*‐value less than 0.05 was considered statistically significant.

## 3 Results

### 3.1 Genome‐Wide Identification of circFOXK2‐DNA Interaction

CircRNA circFOXK2, which is formed by the back‐splicing of exons 2 and 3 of Forkhead box protein K2 (FOXK2), was frequently upregulated in many cancers for tumor growth and metastasis [27, 28, 29]. Our previous study revealed the upregulated circFOXK2 in PDAC tumors [6]. Here, we investigated the clinical significance of high circFOXK2 expression in PDAC by correlating circFOXK2 expression level with clinicopathological characteristics. Male PDAC patients showed increased circFOXK2 levels in PDAC tumors (Table 1). The upregulated circFOXK2 expression level was positively correlated with differentiation (Table 1). Additionally, we previously demonstrated that the upregulated circFOXK2 promoted tumor growth and liver metastasis [6]. Here, we further investigated the role of circFOXK2 in regulating chemotherapy resistance. We assessed the IC_50_ of the common PDAC chemotherapy drug gemcitabine after circFOXK2 knockdown in PDAC cells. CircFOXK2 knockdown in PDAC cells increased response to gemcitabine (Figure 1A and Figure S1A). Collectively, our results suggested the importance of the upregulated circFOXK2 in PDAC progression and chemotherapy resistance.

**FIGURE 1:**
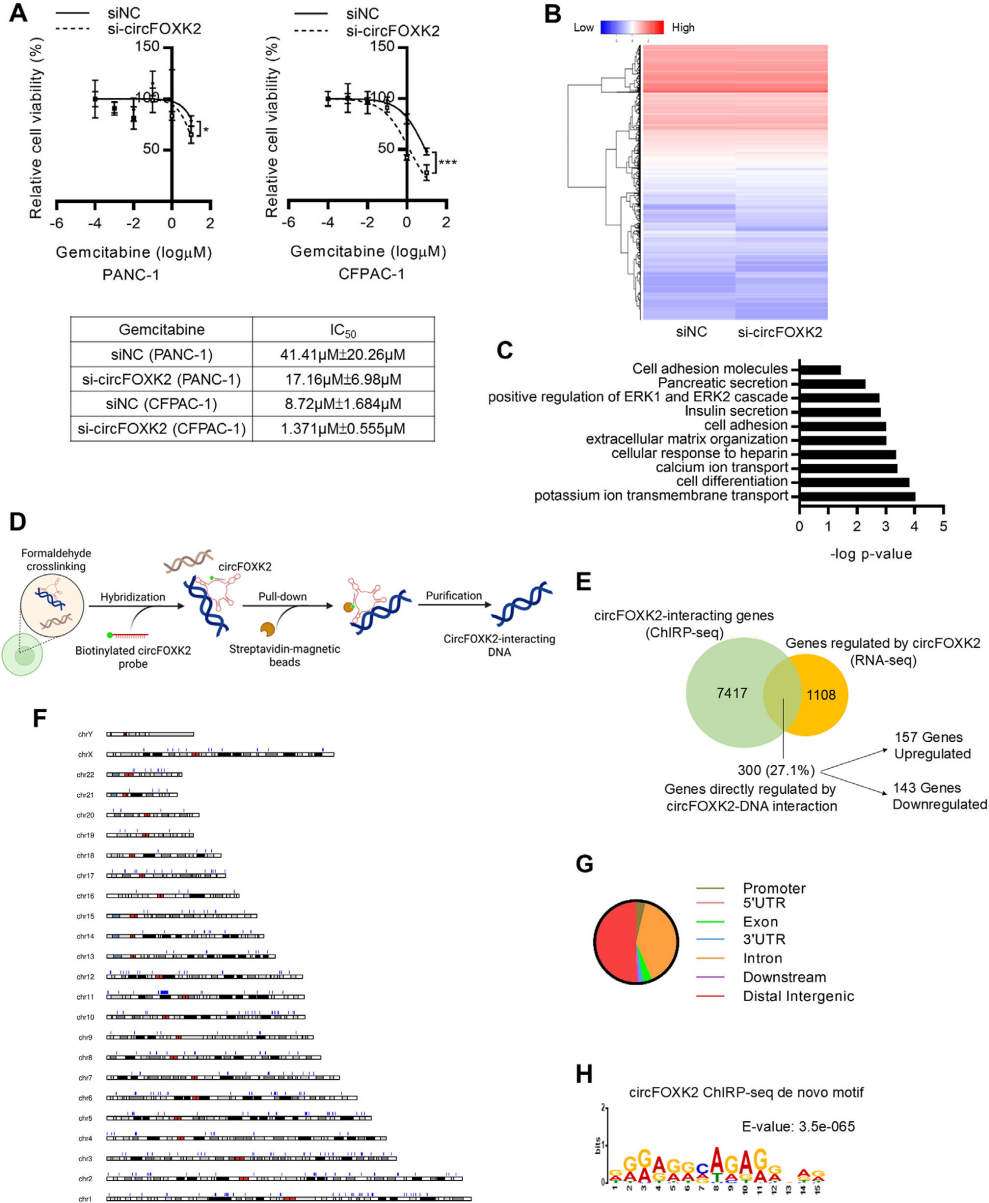
Genome‐wide identification of circFOXK2‐DNA interaction in PDAC cells. (A) CircFOXK2 knockdown sensitized PDAC cells to gemcitabine (*n* = 5 per group). (B) Heatmap for gene expression profile of circFOXK2‐regulating genes. CircFOXK2 was knocked down in PANC‐1 cells, followed by transcriptome profiling by RNA‐seq. (C) GO and KEGG pathway enrichment analysis were performed to examine the importance of circFOXK2‐regulating genes in biological processes. (D) Schematic diagram of the circFOXK2‐specific ChIRP assay followed by DNA sequencing for identifying circFOXK2‐interacting genes in PANC‐1 cells. Biotin‐labeled circFOXK2 probe was used to capture circFOXK2‐chromatin complexes, which were subsequently purified by streptavidin beads. Created in BioRender.com. (E) Venn diagram for overlapping genes with enrichment of circFOXK2 peak and differentially expressed genes after circFOXK2 knockdown in PANC‐1 cells. (F, G) Peak distribution of the circFOXK2‐DNA interaction across (F) chromosomes and (G) genomic locations. (H) Motif analysis of the circFOXK2‐interacting DNA. Data represent mean ± SD (^*^ *p* < 0.05; ^***^ *p* < 0.001).

To reveal its critical molecular mechanistic roles in regulating gene expression, we performed RNA‐sequencing (RNA‐seq) after circFOXK2 knockdown in PANC‐1 cells (Figure 1B). We identified 1,408 circFOXK2‐regulating genes with important roles in many biological processes, including cell differentiation, adhesion, and metabolism (Figure 1C). Emerging studies demonstrated the novel mechanistic roles of circRNAs through DNA interactions for recruiting epigenetic modifiers to the target gene and subsequently transcription regulation [30]. Therefore, we performed chromatin isolation by RNA purification‐sequencing (ChIRP‐seq), which captured the interactions between circFOXK2 and chromatin, and identified 7400 circFOXK2‐interacting genes (Figure 1D). We then overlapped ChIRP‐seq and RNA‐seq datasets for identifying the functional circFOXK2‐DNA interactions and revealed 300 circFOXK2‐regulated genes under direct circRNA‐DNA interaction (Figure 1E and Table S3). We subsequently examined the peak coverage over chromosomes. CircFOXK2‐DNA interaction was observed across chromosomes, in which chromosome 1 harbored the highest frequency (11.1%), followed by chromosome 2 (9.7%) and chromosome 11 (9.4%) (Figure 1F). Analyzing the peak distribution with genomic locations revealed that most circFOXK2 binding peaks were located at the intergenic regions (50.2%), gene bodies (45.1%), and promoters (3.7%) (Figure 1G), with a conserved circFOXK2‐binding motif (Figure 1H), suggesting a global role with high sequence specificity for circFOXK2 in gene regulation.

### 3.2 CircFOXK2 Activates PDAC‐Associated Gene Expression by Sequence‐Specific circRNA‐DNA Interaction

We then investigated the importance of circFOXK2‐DNA interaction in regulating gene expression by selecting a panel of circFOXK2‐interacting genes with different genomic binding regions: trio rho guanine nucleotide exchange factor (TRIO) (intragenic), laminin subunit alpha 4 (LAMA4) (intragenic), brain enriched myelin associated protein 1 (BCAS1) (promoter), thymosin beta 15B (TMSB15B) (promoter), cytochrome P450 26A1 (CYP26A1) (intergenic), and t‐complex 11 (TCP11) (intergenic). We first performed a ChIRP assay to confirm the circFOXK2‐binding to downstream target genes in PANC‐1 cells (Figure 2A). At the same time, circFOXK2 knockdown by back‐splicing junction‐specific siRNAs decreased its binding to them (Figure S1B), and subsequently downregulated their expressions in PDAC cells (Figure 2B,C). CircFOXK2 knockdown in mice PDAC xenografts consistently showed the downregulated target genes (Figure 2D). To reveal the clinical significance of circFOXK2 in regulating the expression of interacting targets, we first examined their expression in pancreatic cancer using the TCGA PAAD datasets. TRIO, LAMA4, TMSB15B, BCAS1, CYP26A1, and TCP11 were frequently upregulated in pancreatic cancer (Figure S3A). Subsequently, we validated their upregulation in our in‐house cohort of PDAC tumor samples and correlated their expressions in PDAC primary tumors by immunohistochemistry (IHC) staining. The upregulated TRIO, LAMA4, BCAS1, TMSB15B, CYP26A1, and TCP11 in PDAC primary tumors showed a positive correlation to circFOXK2 expression level (Figure 2E and Figure S3B). Survival analysis using the TCGA PAAD datasets revealed that the upregulated TRIO and LAMA4 were frequently linked to poor prognosis (Figure S3C). Importantly, the circFOXK2 downstream targets frequently promote cancer progression [31, 32, 33, 34]. Here, we confirmed that TRIO and LAMA4 knockdown in PDAC cells inhibited cancer cell growth, migration, and invasion (Figures S4 and S5). Additionally, the knockdown of circFOXK2 downstream targets could rescue the circFOXK2 oncogenic ability in HPDE cells (Figure S6). These results confirmed the upregulated oncogene expression in promoting PDAC progression under circFOXK2 regulation.

**FIGURE 2:**
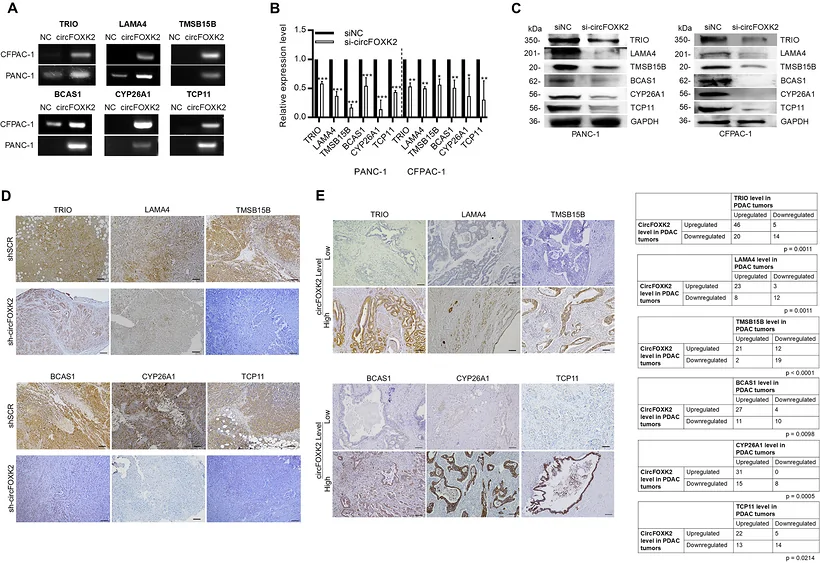
CircFOXK2 regulates TRIO, LAMA4, TMSB15B, BCAS1, CYP26A1, and TCP11 expression in PDAC. (A) ChIRP assay confirmed that circFOKX2 bound to the target genes' DNA in PDAC cells. (B, C) (B) qRT‐PCR and (C) immunoblotting showed that the circFOXK2 knockdown inhibited target gene expression in PDAC cells. (D) Immunohistochemistry staining showed that circFOXK2 knockdown in mice PDAC xenograft (*n* = 7 per group) inhibited target genes. Mouse xenograft from the previous study was analyzed [6]. (E) A positive correlation between circFOXK2 and its target genes' expression was observed in PDAC tumors. Data represent mean ± SD from at least three independent experiments (^*^ *p* < 0.05; ^**^ *p* < 0.01; ^***^ *p* < 0.001).

### 3.3 Single‐Cell ATAC‐seq in PDAC Cells Reveals the Roles of circFOXK2 in Regulating Gene Transcription

The intratumoral heterogeneity of PDAC plays an important role in cancer development and progression. To investigate the circFOXK2‐DNA regulatory pathways in intratumoral heterogeneity at the chromatin level for active gene expression, we performed a single‐cell sequencing assay for transposase‐accessible chromatin (scATAC‐seq) analysis using the publicly available dataset in PANC‐1 cells [10]. After initial quality control and filtering, we obtained 6070 cells with 115 030 features (represented by unique molecular identifier, UMI) by principal component analysis (PCA). Through uniform manifold approximation and projection (UMAP), we obtained a single‐cell chromatin accessibility map of five subclusters with the distinct shape of the cell population (Figure 3A). We analyzed the genomic distribution of accessible regions in PDAC single‐cells. The accessible sites were mainly located in the promoter (37.84%) and introns (33.99%), followed by distal intergenic (23.8%) and exons (2.28%) (Figure 3B). We aligned the cells in each cluster through pseudotime trajectory analysis, which reflects the dynamic process of PDAC tumorigenesis based on the gene activities, using Monocle3. We found that cluster 2, with lower transcription activities, was identified as an early state. In contrast, cluster 0 and cluster 3, with increased transcriptional activities, were connected to a later state (Figure 3C). We then annotated each cluster based on its differential transcriptional activities. Compared with the early clusters, the late cancer cells revealed 7070 differential accessibility peaks (from 6,318 genes). KEGG pathways and GO analysis consistently indicated that late clusters harbored transcription‐active genes for pancreatic cancer, cell growth, and cell migration; while the early clusters showed the feature of increased Ras signaling activity, stem cell division, and stem cell maintenance (Figure 3D), suggesting the PDAC intratumoral heterogeneity along the malignant continuum from stem‐like properties for cancer progression.

**FIGURE 3:**
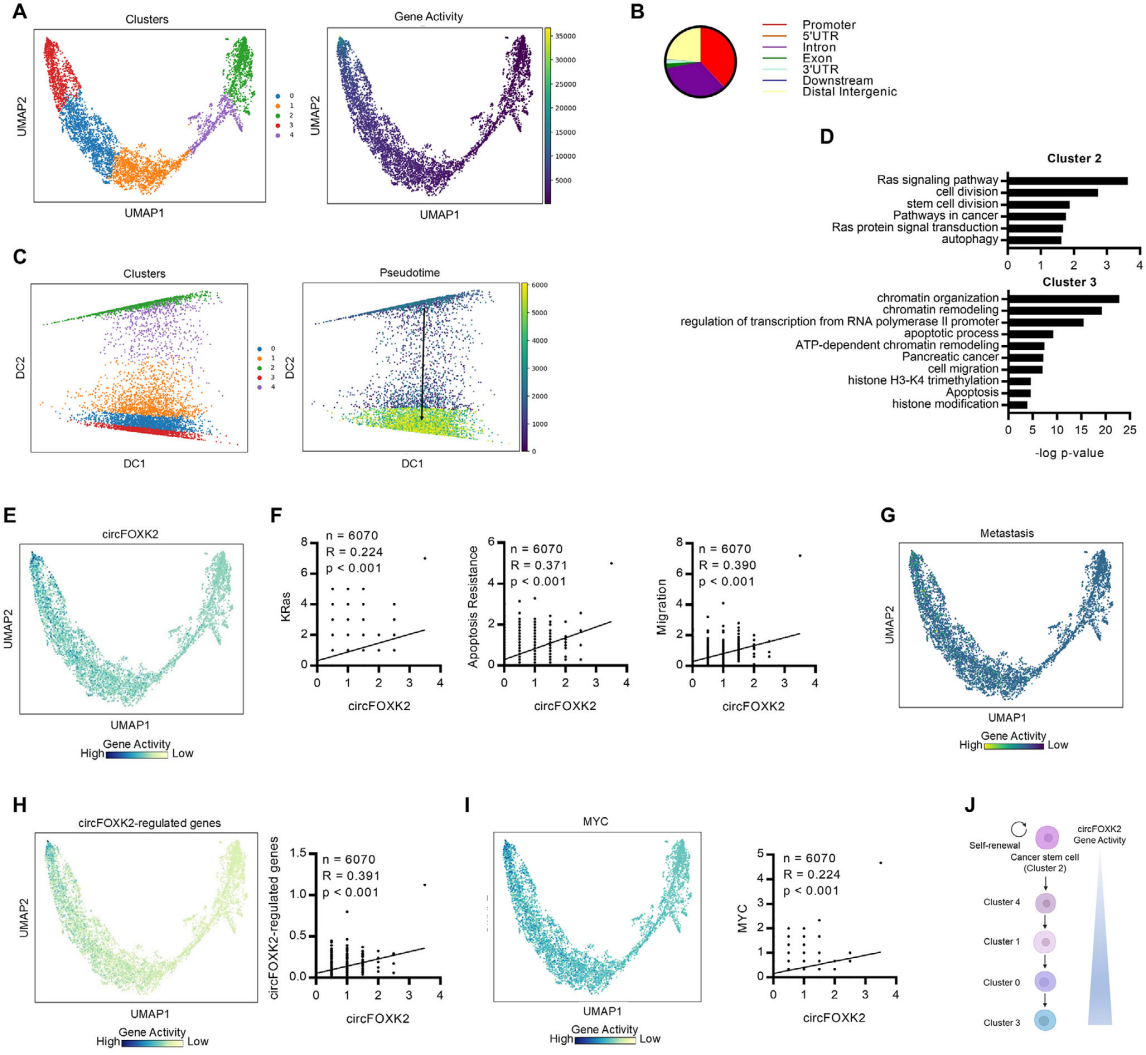
Single‐cell ATAC‐seq in PDAC cells reveals the roles of circFOXK2 in regulating gene transcription. (A) 2D Uniform manifold approximation and projection (UMAP) of the PANC‐1 scATAC‐seq dataset labeled and colored by cancer sub‐cluster. (B) Peak distribution of the identified accessible sites. (C) pseudotime trajectory analysis identified the dynamic process of PDAC tumorigenesis and differentiation. (D) GO and KEGG pathway analysis of the differential peaks in each PDAC sub‐cluster. (E) CircFOXK2 gene activity was increased in cluster 3. (F) A positive correlation between circFOXK2, Kras, apoptosis‐resistance gene, and migration gene activities in PDAC cells. (G) UMAP plot of the metastatic ability in each PDAC subcluster. Cluster 3 with increased circFOXK2 gene activity showed an increased metastatic ability. (H,I) UMAP plot of the gene activities of (H) circFOXK2‐regulated genes and (I) MYC in each PDAC subcluster. A positive correlation was observed with circFOXK2. (J) Schematic diagram showing the PDAC tumorigenesis with increasing circFOXK2 gene activity. Created in BioRender.com.

To reveal the circFOXK2‐DNA interaction regulatory mechanisms regulating the heterogeneous chromatin accessibility in PDAC, we first examined the circFOXK2 gene activity in each PANC‐1 subcluster. Cancer cells with increased histone methylation, chromatin remodeling, and gene transcription harbored increased circFOXK2 gene activities (Figure 3E), indicating that the upregulated circFOXK2 during PDAC development is linked to a more aggressive lineage with enhanced epigenetic and transcriptional activities. Particularly, we correlated Kras, migration‐related genes, apoptosis‐resistance gene, and circFOXK2 gene activity at the single‐cell level and observed positive correlations with the upregulated circFOXK2 gene activity (Figure 3F). Furthermore, we examined the proportion of metastatic cells in each sub‐cluster. Clusters 0 and 3 with upregulated circFOXK2 showed an increase in metastatic ability (Figure 3G), which was consistent with our previous in vitro and in vivo studies in demonstrating the importance of circFOXK2 in PDAC apoptosis‐resistance, growth, and metastasis [6].

Notably, these clusters consistently showed evaluated gene activities of circFOXK2‐regulatory downstream targets, which were identified by the bulk RNA‐seq (Figure 3H). To identify the factors regulating circFOXK2 expression, we performed the transcription factor binding analysis of the circFOXK2 promoter using the ENCODE and ChIP Enrichment Analysis (ChEA) (Figure S7A) [10, 35]. ChIP‐seq analysis using the ENCODE datasets confirmed the binding of MYC and FOS to the circFOXK2 promoter (Figure S7B). Subsequently, we examined the gene activities of the transcription factors at the single‐cell level in PDAC. Clusters 0 and 3 showed an increased *MYC* gene activity with a positive correlation with circFOXK2 (Figure 3I and Figure S7C), suggesting that circFOXK2 was potentially regulated by MYC in PDAC. Collectively, the positive correlation between circFOXK2 and its downstream target gene activities in the PDAC cells suggested the importance of circFOXK2 in regulating chromatin accessibility in PDAC, contributing to the cancer heterogeneity for promoting PDAC progression (Figure 3J).

### 3.4 CircFOXK2 Interacts With MLL Inhibitor PPIE

We then investigated the detailed mechanism by which circFOXK2 interacted with DNA targets at different genomic regions to regulate their expression. We previously performed a circFOXK2‐specific pulldown assay to obtain circFOXK2‐interacting proteins, followed by mass spectrometry analysis (Figure 4A) [6]. KEGG pathway and GO analysis of the circFOXK2‐interacting proteins revealed their important roles in epigenetics, chromatin structure, and gene transcription. We particularly focused on PPIE, also known as cyclophilin 33, which is an important MLL1, also known as lysine methyltransferase 2A (KMT2A), regulator for H3K4me. Simultaneously, RNA immunoprecipitation assay demonstrated the circFOXK2‐PPIE interaction in PDAC cells (Figure 4B and Figure S8A). Electrophoretic mobility shift assay (EMSA) using in vitro transcribed circFOXK2 and purified PPIE gave consistent results (Figure 4C), indicating the circFOXK2‐PPIE interaction.

**FIGURE 4:**
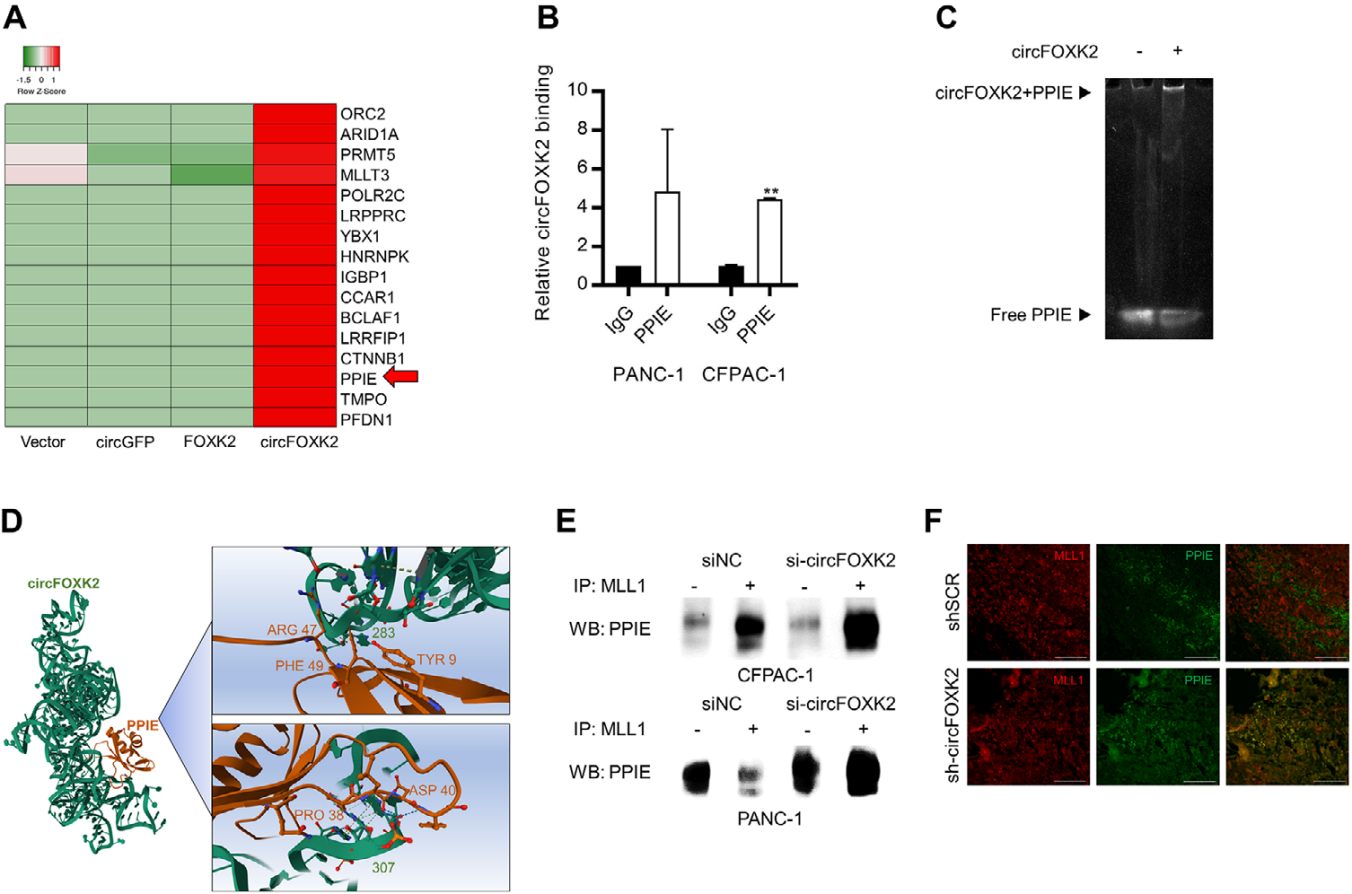
CircFOXK2‐PPIE interaction through RRM disrupts PPIE‐MLL1 interaction. (A) CircFOXK2‐specific pulldown assay was performed to identify circFOXK2‐interacting proteins. A heatmap was constructed to show the abundance of the identified chromatin and transcription‐regulating proteins. Previously published data [6] were analyzed. (B) RNA immunoprecipitation assay was performed to validate the circFOXK2‐PPIE interaction in PDAC cells. (C) Electrophilic mobility shift assay showed the in vitro interaction of circFOXK2 with PPIE. (D) Three‐dimensional computational modeling identified the circFOXK2‐PPIE binding sites. The AU‐rich binding motif of circFOXK2 interacted with PPIE RRM. (E) Co‐immunoprecipitation assay showing the decreased MLL1‐PPIE interaction after circFOXK2 knockdown in PDAC cells. (F) Immunofluorescent images for PPIE (green) and MLL1 (red) location after circFOXK2 knockdown in mice CFPAC‐1 subcutaneous xenograft (*n* = 7 per group). Mouse xenograft from the previous study was analyzed [6]. Data represent mean ± SD from at least three independent experiments (^**^ *p* < 0.01).

PPIE consists of a C‐terminal cyclophilin domain and an N‐terminal RNA‐recognition motif (RRM), which binds to the MLL1 zinc finger PHD3 domain to prevent target genes from binding [36, 37]. At the gene‐activating regions, AU‐rich RNAs compete with MLL1 for the PPIE RRM binding, and eventually displace PPIE from MLL1 to remove the gene‐inhibitory effect [38]. However, the exact RNA involved in this PPIE displacement is still unknown. Since we revealed circFOXK2‐PPIE interaction in PDAC, which showed an upregulated H3K4me level (Figure S8B), we hypothesized that the upregulated H3K4me in PDAC was contributed by circFOXK2‐mediated PPIE displacement.

We performed HDOCK analysis to investigate the detailed structure of circFOXK2‐PPIE interaction. Three‐dimensional (3D) computational modeling revealed that circFOXK2 contained multiple helical structures, formed by the regional intramolecular complementary base pairing of the single‐stranded circular structure (Figure 4D). This unique structure generated a docking pocket, which fits the RRM domain of PPIE. Notably, the interaction pocket of circFOXK2 contained two AU‐rich binding motifs (AAUAAUAA) at residues 283 and 307, facilitating the binding of PPIE via its amino acids TYR9, ARG47, PHE49, PRO38, and ASP40. This interaction model was confirmed by RBPmap analysis, in which the circFOXK2 harbored the AU‐rich PPIE‐binding motif (Figure S8C). To explore the structural importance of circFOXK2 to PPIE binding, the PPIE‐binding motif on circFOXK2 was disrupted by mutagenesis for the subsequent circFOXK2‐pulldown assay, which showed the inhibited circFOXK2‐PPIE interaction (Figure S8C,D). Collectively, our data suggested that circFOXK2 may bind to PPIE to remove the MLL1 inhibitory effect.

We then knocked down circFOXK2, followed by a co‐immunoprecipitation assay to examine the effect on PPIE‐MLL1 interaction. The PPIE‐binding to the MLL1 was increased after circFOXK2 knockdown in PDAC cells (Figure 4E and Figure S8E). Using the mouse PDAC xenograft with circFOXK2 knockdown [6], we performed immunofluorescent staining of PPIE and MLL1 to visualize the intracellular co‐localization. Consistently, intracellular co‐localization of PPIE and ML1 was increased after circFOXK2 knockdown (Figure 4F), confirming that circFOXK2 knockdown facilitated PPIE‐MLL1 interaction. Subsequently, mutating this PPIE‐binding motif on circFOXK2 could promote PPIE‐MLL1 interaction (Figure S8F), suggesting that the loss of circFOXK2‐PPIE interaction contributed to the increased PPIE‐MLL1 interaction.

### 3.5 CircFOXK2 Sequesters PPIE From MLL1 for Promoting H3K4 Methylation and Gene Transcription

After confirming the importance of circFOXK2 in disrupting PPIE‐MLL1 interaction, we further examined the circFOXK2‐mediated MLL1 activities. After circFOXK2 knockdown, there was a generally decreased H3K4me level in PDAC cells and mice xenograft (Figure 5A,B and Figure S9), indicating the increased MLL1 inhibitory effect. Since the PPIE expression level is unchanged after circFOXK2 knockdown (Figure 5A), the decrease in MLL1 activity by circFOXK2 was not due to altering PPIE expression. Furthermore, either mutating the PPIE‐binding motif on circFOXK2 or knocking down MLL1 could subsequently rescue the oncogenic abilities of circFOXK2 in HPDE cells with a low endogenous circFOXK2 expression level (Figure S10). These suggested the roles of circFOXK2‐PPIE in promoting MLL1 activities and PDAC progression.

**FIGURE 5:**
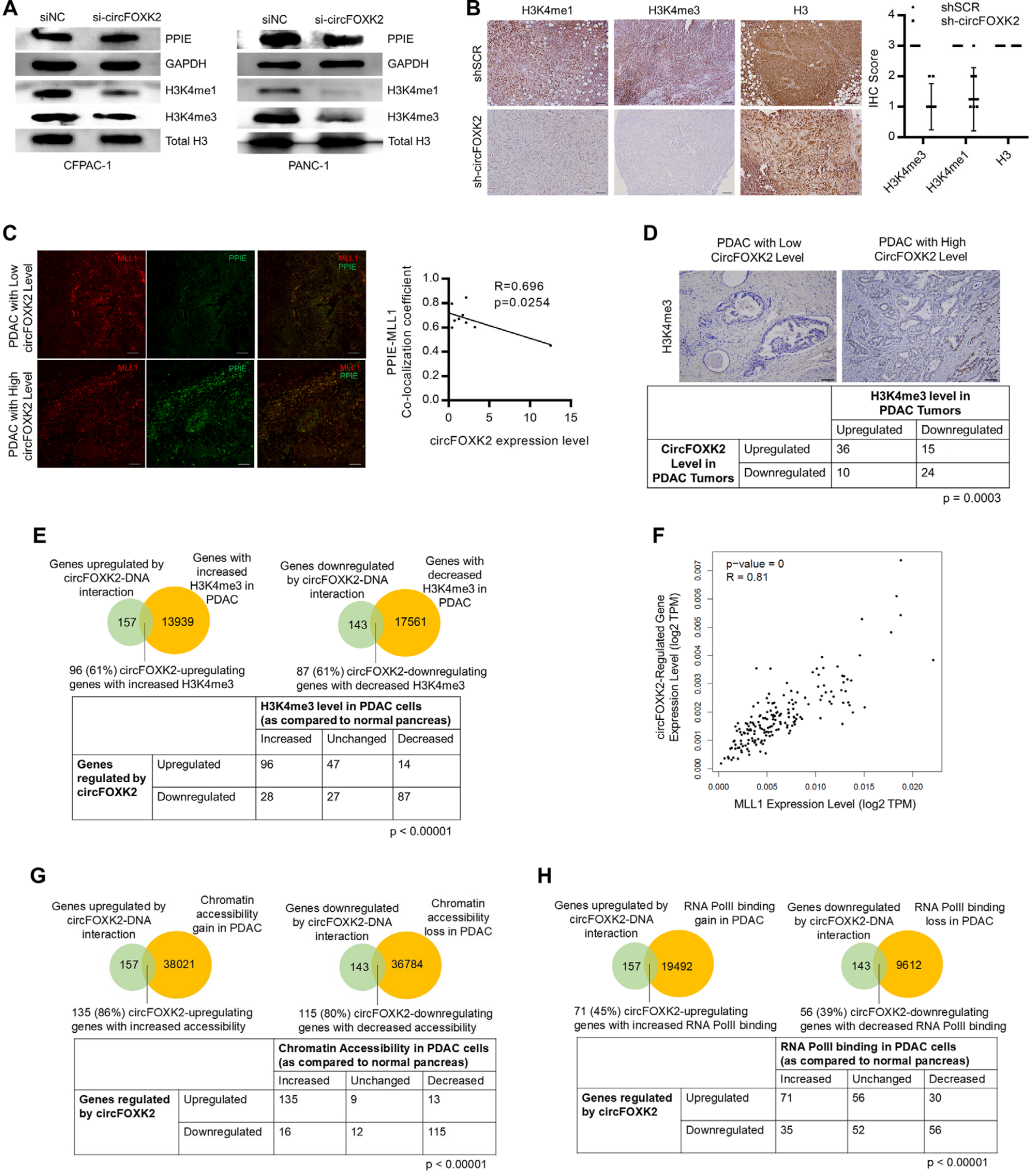
Direct circFOXK2‐DNA interaction promotes H3K4me. (A, B) The total histone H3K4me1 and H3K4me3 levels in PDAC (A) cells and (B) mice xenografts (*n* = 7 per group) were decreased after circFOXK2 knockdown. Mouse xenograft from the previous study was analyzed [6]. (C) Immunofluorescent images for PPIE (green) and MLL1 (red) location in PDAC primary tumors with differential circFOXK2 expression. PDAC tumors with upregulated circFOXK2 showed a decrease in MLL1‐PPIE co‐localization. (D) The total histone H3K4me3 levels in PDAC primary tumors with positive correlation to circFOXK2 expression. (E) Venn diagram for overlapping genes under circFOXK2 regulation with differentially H3K4me3 levels. Genes regulated under circFOXK2‐DNA interaction were identified by ChIRP‐seq and RNA‐seq analysis (Figure 1D). Genes with differential H3K4me3 levels in PDAC were obtained from comparing ChIP‐seq data from PDAC and the normal pancreas. (F) A positive correlation between circFOXK2‐interacting genes and MLL1 expression in PDAC tumors. Transcriptomic analysis of the PDAC tumors was performed by GEPIA2 using TCGA PDAC dataset. (G, H) Venn diagram for overlapping genes under circFOXK2 regulation with differentially (G) nuclear accessibility and (H) RNA PolII binding in PDAC. Genes regulated under circFOXK2‐DNA interaction were identified by ChIRP‐seq and RNA‐seq analysis (Figure 1D). Genes with differential nuclear accessibility and RNA PolII binding in PDAC were obtained from comparing DNase‐seq and ChIP‐seq data from PDAC and the normal pancreas. Data represent mean ± SD from at least three independent experiments.

To reveal the clinical significance of this circFOXK2‐mediated PPIE‐MLL1 interaction, we examined the PPIE and MLL1 co‐localization in PDAC tumors by immunofluorescent staining with differentially expressed circFOXK2. PDAC tumors with upregulated circFOXK2 showed decreased PPIE‐MLL1 colocalization, as quantitatively revealed by the Mander's overlap coefficient (Figure 5C). Consistently, measuring H3K4me3 level in PDAC tumors revealed a positive correlation with circFOXK2 expression level but a negative correlation with PPIE‐MLL1 interaction (Figure 5D). The circFOXK2‐interacting genes showed a positive association with the MLL1 expression and H3K4me3 level in PDAC tumors (Figure 5E,F). Furthermore, the H3K4me3 level is reportedly important for promoting gene transcription by forming euchromatin and enhancing gene transcription [9, 39]. Therefore, we also examined the changes in chromatin structure in circFOXK2‐interacting genes in PDAC by analyzing DNase‐seq and ChIP‐seq datasets. The increased H3K4me3 level in PDAC tumors is frequently linked with the increased chromatin accessibility, which subsequently facilitated the gene transcription process, as indicated by the increased RNA Polymerase II (RNA PolII) binding (Figure S11). Consistently, circFOXK2‐interacting gene expression showed a positive correlation with the increased chromatin accessibility and RNA PolII binding for promoting their expression (Figure 5G,H). Altogether, these results suggested that circFOXK2 bound to PDAC‐associated genes to promote transcription by enhancing MLL1‐mediated H3K4me in PDAC.

### 3.6 CircFOXK2 Promotes Transcription Elongation by Facilitating Intragenic H3K4me3

We then investigated how specific genes *TRIO* and *LAMA4* were regulated by circFOXK2 at intragenic binding in PDAC by integrating H3K4me3 (ChIP‐seq) and chromatin accessibility (DNase‐seq). Given that circFOXK2 promoted gene‐activating H3K4me3 by removing the PPIE‐inhibitory effects on MLL1, we first examined the H3K4me3 and DNase‐seq profile on *TRIO* and *LAMA4* genes. H3K4me3 level and chromatin accessibility on the *TRIO* and *LAMA4* genes were increased (Figure 6A). MLL1 knockdown downregulated H3K4me3 level, chromatin accessibility, and subsequently TRIO and LAMA4 expression (Figures S12 and S13). Conversely, PPIE knockdown upregulated H3K4me3 level, chromatin accessibility, and subsequently TRIO and LAMA4 expression (Figure S14). Importantly, circFOXK2 knockdown promoted PPIE binding but decreased MLL1 binding and H3K4me3 level on the *TRIO* and *LAMA4* genes (Figure 6B,C). This decreased H3K4me3 level after circFOXK2 knockdown could inhibit chromatin accessibility (Figure 6D). Also, RNA PolII and its phosphorylated‐serine‐2 (RNA PolII Ser2) level, which is the indicator of active transcription elongation process [40], on the *TRIO* and *LAMA4* genes were subsequently decreased (Figure 6E,F). These eventually inhibited gene transcription processes after circFOXK2 knockdown in PDAC cells (Figure 6G).

**FIGURE 6:**
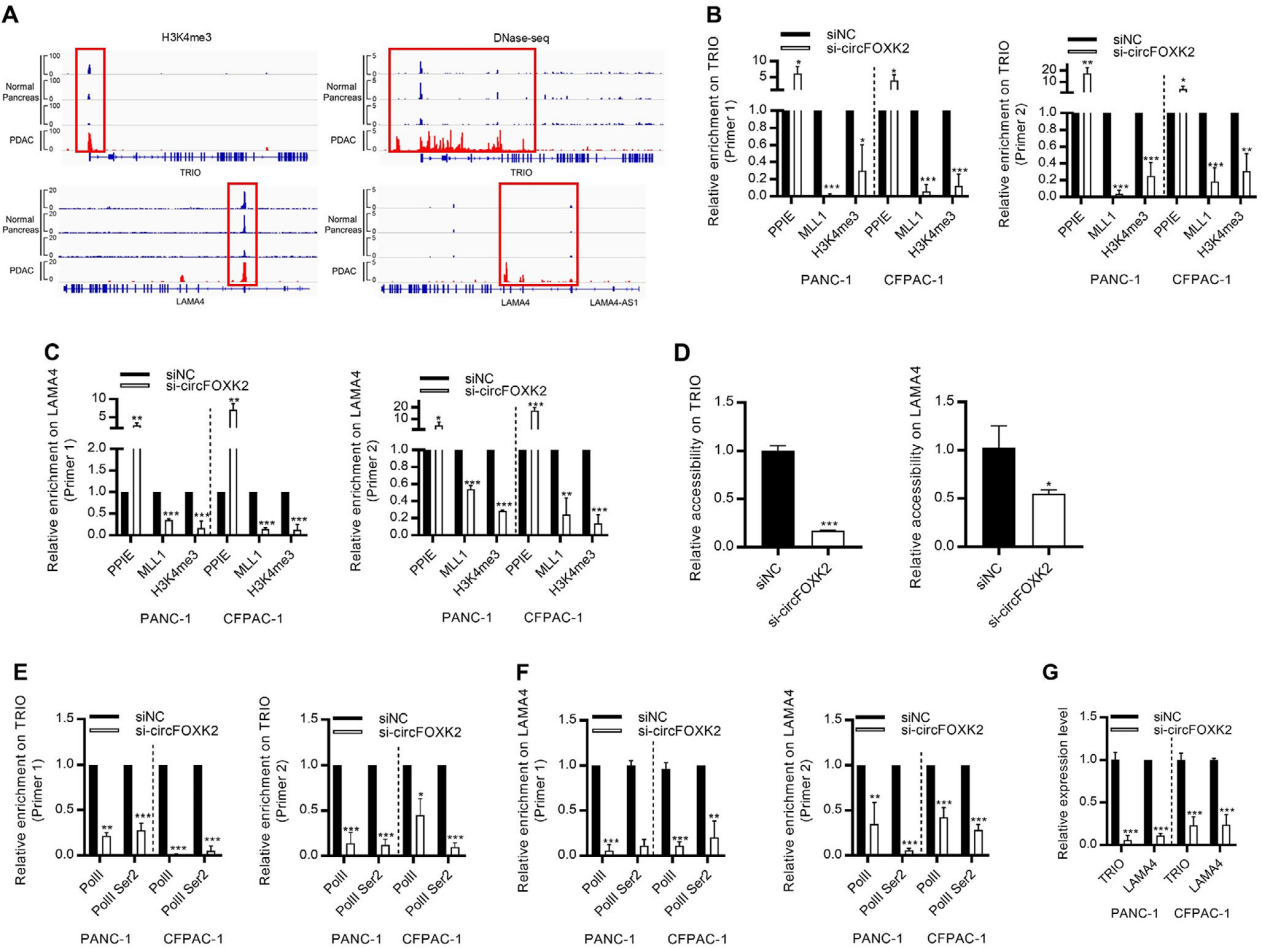
CircFOXK2 promotes transcription elongation by H3K4me3 at the intragenic region. (A) Track view showing the H3K4me3 ChIP‐seq and DNase‐seq signals of *TRIO* and *LAMA4* genes in PDAC, compared with the normal pancreas. (B and C) CircFOXK2 knockdown in PDAC cells increased PPIE binding but decreased MLL1 binding and H3K4me3 level at (B) *TRIO* and (C) *LAMA4*. Two primers were used in the ChIP‐PCR. (D) CircFOXK2 knockdown in PDAC cells reduced chromatin accessibility of *TRIO* and *LAMA4*. (E, F) CircFOXK2 knockdown in PDAC cells reduced RNA PolII and serine‐2 phosphorylation levels of (E) *TRIO* and (F) *LAMA4*. Two primers were used in the ChIP‐PCR. (G) CircFOXK2 knockdown in PDAC cells reduced the transcription elongation efficiency of *TRIO* and *LAMA4*, as revealed by the nuclear run‐on assay. Data represent mean ± SD from at least three independent experiments (^*^ *p* < 0.05; ^**^ *p* < 0.01; ^***^ *p* < 0.001).

### 3.7 CircFOXK2 Binds to the Promoter and Enhancer for Gene‐Activating H3K4me

Apart from facilitating transcription elongation, MLL1‐mediated H3K4me3 at the promoter is responsible for gene activation. Compared with the normal pancreas, H3K4me3 at the *TMSB15B* and *BCAS1* promoter was increased in PDAC (Figure 7A). MLL1 knockdown in PDAC cells decreased H3K4me3 level at the *TMSB15B* and *BCAS1* promoter and subsequently downregulated their expression (Figure S13). Alternatively, PPIE knockdown in PDAC cells upregulated TMSB15B and BCAS1 expression (Figure S14). Importantly, circFOXK2 knockdown decreased MLL1 binding to *TMSB15B* and *BCAS1* promoter and led to a decrease in H3K4me3 level (Figure 7B,C). This eventually inhibited TMSB15B and BCAS1 expression in PDAC (Figure 2B–D).

**FIGURE 7:**
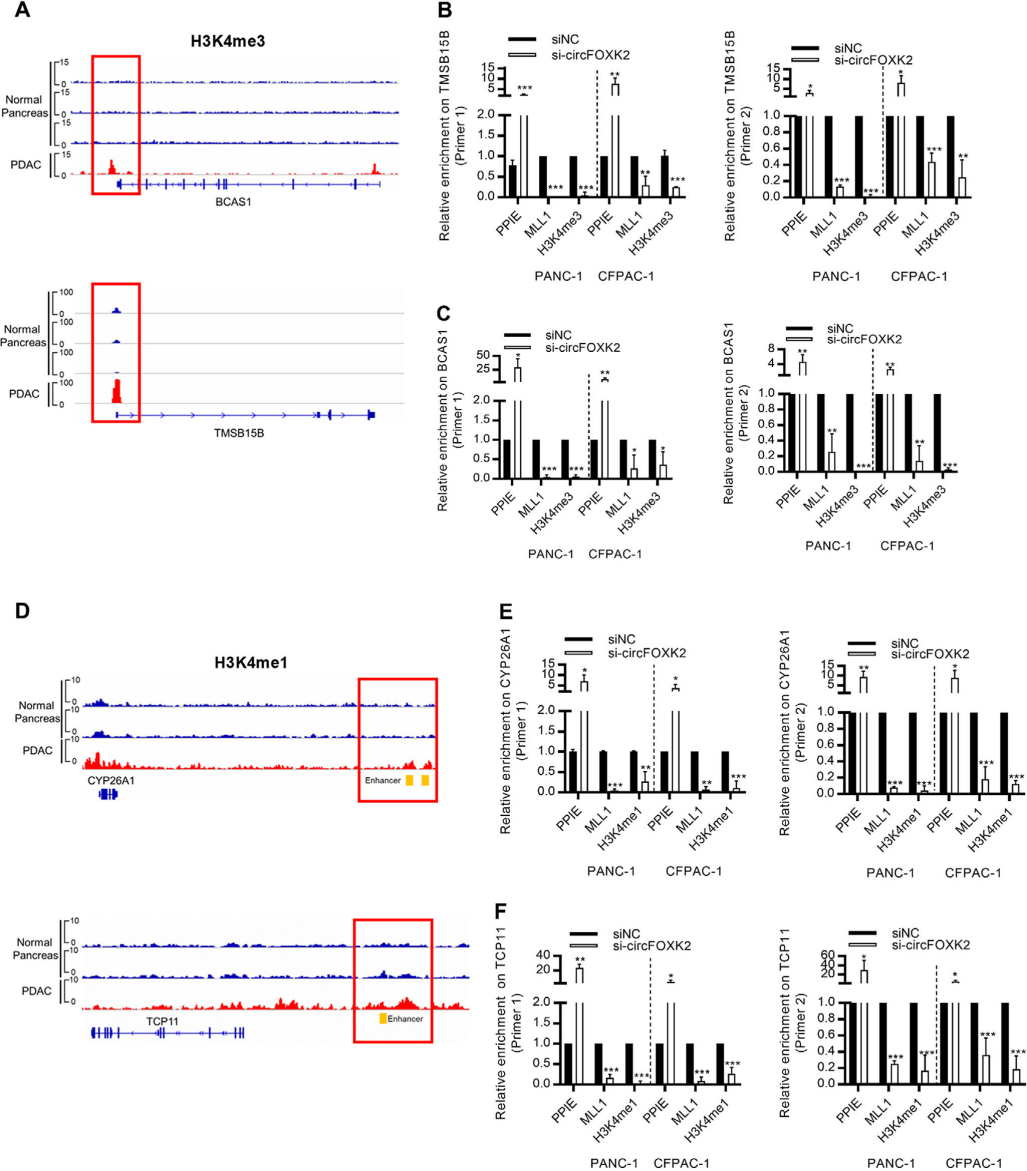
CircFOXK2 activates gene expression by H3K4me at the promoter and enhancer. (A) Track view showing the H3K4me3 ChIP‐seq signals of TMSB15B and BCAS1 gene in PDAC, and compared with the normal pancreas. (B, C) CircFOXK2 knockdown in PDAC cells increased PPIE binding and decreased MLL1 binding and H3K4me3 level at (B) *TMSB15B* and (C) *BCAS1* promoter. Two primers were used in the ChIP‐PCR. (D) Track view showing the H3K4me1 ChIP‐seq signals of *CYP26A1* and *TCP11* gene in PDAC, and compared with the normal pancreas. Enhancer information was obtained from the UCSC genome browser. (E, F) CircFOXK2 knockdown in PDAC cells increased PPIE binding and decreased MLL1 binding and H3K4me1 level at (E) *CYP26A1* and (F) *TCP11* enhancer. Two primers were used in the ChIP‐PCR. Data represent mean ± SD from at least three independent experiments (^*^ *p* < 0.05; ^**^ *p* < 0.01; ^***^ *p* < 0.001).

Although MLL1 is well‐recognized for H3K4me3 at promoters and intragenic regions, it is also involved in catalyzing H3K4me1 and H3K4me3 at enhancers [41, 42, 43]. Over half of the circFOXK2‐binding sites were in the intergenic regions with increased H3K4me1 and H3K4me3 levels. Compared with the normal pancreas, H3K4me1 at the *CYP26A1* and *TCP11* enhancer was increased in PDAC (Figure 7D). Given that circFOXK2 functioned as the MLL1 activator, the circFOXK2‐DNA interaction at intergenic regions may contribute to active enhancers for gene activation in PDAC. MLL1 knockdown inhibited CYP26A1 and TCP11 expressions by decreasing H3K4me1 level at enhancer (Figure S13), while PPIE knockdown promoted CYP26A1 and TCP11 expression (Figure S14). Importantly, similar to the promoters and intragenic regions, MLL1 binding at enhancers after circFOXK2 knockdown was decreased with a subsequent reduction in H3K4me1 (Figure 7E,F), resulting in CYP26A1 and TCP11 downregulation (Figure 2B–D). Taken together, our results suggested the importance of circFOXK2 in MLL1‐mediated H3K4me for gene activation and transcription elongation.

## 4 Discussion

CircRNAs are characterized by their covalently closed circular structure. Functioning as miRNAs sponge is the first identified mechanistic role of circRNAs in regulating gene expression. Notably, emerging studies identified additional critical roles of circRNAs in epigenetics through protein interaction, RNA and DNA binding, as many long non‐coding RNAs (lncRNAs) [15]. LncRNA HOTTIP functions as a scaffold for recruiting MLL/WDR5 complex to gene promoters for activating H3K4me3 [9]. LncRNA ecCEBPA sequesters DNA methyltransferase DNMT1 from chromatin for gene hypomethylation and transcription activation [44]. Similarly, several circRNAs were reportedly bound to DNA for epigenetic modifications and gene transcription regulation. CircRNA FECR1 recruits DNA demethylase TET1 to the FLI1 promoter for removing gene silencing DNA methylation [33]. CircEIF3J and circPAIP2 interact with U1 snRNA and RNA PolII at gene promoter and first exon for transcription initiation [45]. CircANKRD52 interacts with RNA PolII phosphor‐Ser2 for enhancing transcription elongation [46]. These discoveries of novel mechanisms suggest the detailed mechanistic roles of circRNAs remain largely unexplored.

We previously identified the upregulated circRNA circFOXK2 with important roles in promoting PDAC growth and metastasis [6]. Here, we found that male PDAC patients with differentiated tumors showed further increased circFOXK2 expression. In addition to PDAC, the upregulated circFOXK2 was found in breast cancer [27], non‐small cell lung cancer [28], oral squamous cell carcinoma [47], and hepatocellular carcinoma for promoting cancer progression [29]. These studies suggest the therapeutic direction in treating multiple cancers through targeting the upregulated circFOXK2. Herein, we performed a genome‐wide, multi‐omics, and single‐cell investigation to reveal the novel mechanism of circFOXK2 in gene regulation by circRNA‐DNA interaction. ChIRP‐seq and transcriptomic profiling after circFOXK2 knockdown reveal the critical roles of circFOXK2 at different genomic locations. Mass spectrometry analysis of the circFOXK2‐interacting proteins identified proteins for gene transcription and epigenetics in PDAC cells. We particularly focused on circFOXK2‐PPIE interaction, in which circFOXK2 removed the PPIE inhibitory effect from MLL1 at the target gene for transcription activation and elongation.

MLL1 is a well‐known epigenetic modifier for gene activating H3K4me in promoter by its SET domain and PHD3 domain [48]. In addition to its well‐recognized role in promoters for activating gene transcription, MLL1 in other genomic locations is also important for gene expression. H3K4me1, which is specifically enriched in the intergenic regions, marks an active enhancer for promoting chromatin remodeling and RNA PolII binding [49, 50]. Broad H3K4me3 peak in the promoter and first exon is required for efficient transcription by facilitating the pause‐release of RNA PolII from the transcription start site [51, 52]. During the transcription process, intragenic H3K4me3 can facilitate the transcription elongation process and gene expression by forming an euchromatin structure [53]. Notably, these gene regulatory mechanisms of MLL1 are highly regulated by an inhibitor PPIE, in which its PPIase domain isomerizes MLL1 for promoting interaction between the RRM domain of PPIE and the PHD3 domain of MLL1 [54]. This eventually disrupts the PHD3‐bromo interface of MLL1 and inhibits H3K4me. Apart from MLL1, other MLL family members, MLL2‐4, also contain the PHD3 domain with high sequence similarity. However, a study on the MLL protein structure found that the MLL2 PHD3 domain did not interact with PPIE [54]. Only MLL1, which harbors the unique Met1606 residue at the interacting site, can be regulated by PPIE. Notably, this PPIE‐MLL1 interaction was reported to be mediated by RNA. Studies demonstrated that RNA bound to the RRM of PPIE, inducing its conformational changes for MLL1 interaction [55, 56]. Also, other AU‐rich RNAs were found to interact with PPIE for removing the MLL1‐inhibitory effect [38, 54]. However, the exact RNA involved in PPIE sequestration is still unknown. In the current study, we found that circFOXK2 harbored this AU‐rich PPIE‐binding motif for forming a regional docking pocket for the RRM domain of PPIE. Importantly, circFOXK2 formed a DNA‐DNA‐RNA triplex through complementary base‐pairing for target gene recognition at different genomic locations. At the target gene, circFOXK2‐PPIE interaction could displace PPIE from MLL1, restoring normal MLL1 confirmation for gene activating H3K4 methylation and increasing gene transcription.

The intratumoral heterogeneity of PDAC plays an important role in cancer development and progression. Single‐cell transcriptomics of cancer cell lines also revealed their heterogeneity, which could be divided into two types: distinct type with spatially discrete subclusters, and continuous type without a clear border between subclusters [57]. These clinically relevant intratumoral heterogeneity determined their stemness, aggressiveness, metastasis, and therapeutic response [58, 59]. In the current study, scATAC‐seq of PANC‐1 cells revealed the continuous subclusters of PDAC cells. Notably, PDAC cell subclusters with upregulated circFOXK2 gene activities were linked to the differentiated state with enhanced epigenetic and transcriptional activities. These cancer subclusters were featured by increased proliferative and metastatic abilities. This was consistent with our previous finding on circFOXK2 in promoting PDAC tumor growth and liver metastasis [6]. Mechanistically, the PDAC cell subclusters at the differentiated state showed an increased gene activities of the circFOXK2‐regulating targets. CircFOXK2 gene activity at the single‐cell level was positively correlated with its downstream target gene expression, suggesting the circFOXK2‐regulatory pathways in regulating PDAC heterogeneity. Particularly, the upregulated circFOXK2 promoted the dysregulated TRIO, LAMA4, TMSB15B, BCAS1, CYP26B1, and TCP11 in PDAC. Upregulated TRIO was frequently observed in many cancers to promote cancer cell migration and invasion [31]. LAMA4, which is an extracellular glycoprotein for cell adhesion and migration, was significantly upregulated in PDAC in promoting liver metastasis [32]. The upregulated TMSB15B promoted cancer cell migration [33]. CYP26A1 upregulation in PDAC promoted cancer cell growth and invasion [34]. Furthermore, we identified MYC as the potential transcription factor regulating circFOXK2 expression in PDAC. PDAC cells at the single‐cell level showed a positive correlation between circFOXK2 and MYC gene activities. ChIP‐seq analysis revealed the binding of MYC to the promoter of circFOXK2. Altogether, the upregulated circFOXK2 by MYC activated oncogene expression by H3K4 methylation through circRNA‐DNA interaction.

## 5 Conclusion

CircFOXK2 played important roles in epigenetics at the enhancer, promoter, and intragenic regions. Mechanistically, circFOXK2 interacted with downstream genes by the sequence complementary, forming a DNA‐DNA‐RNA triplex. At the target sites, circFOXK2 interacted with PPIE through its AU‐rich motif and unique docking pocket, removing the MLL1 inhibitory effects for promoting gene‐activating H3K4 methylation (Figure 8). Our results demonstrate new insights into the functional and mechanistic importance of circRNAs in gene regulation and uncover the novel therapeutic direction targeting circRNA‐DNA interaction in PDAC and other diseases.

**FIGURE 8:**
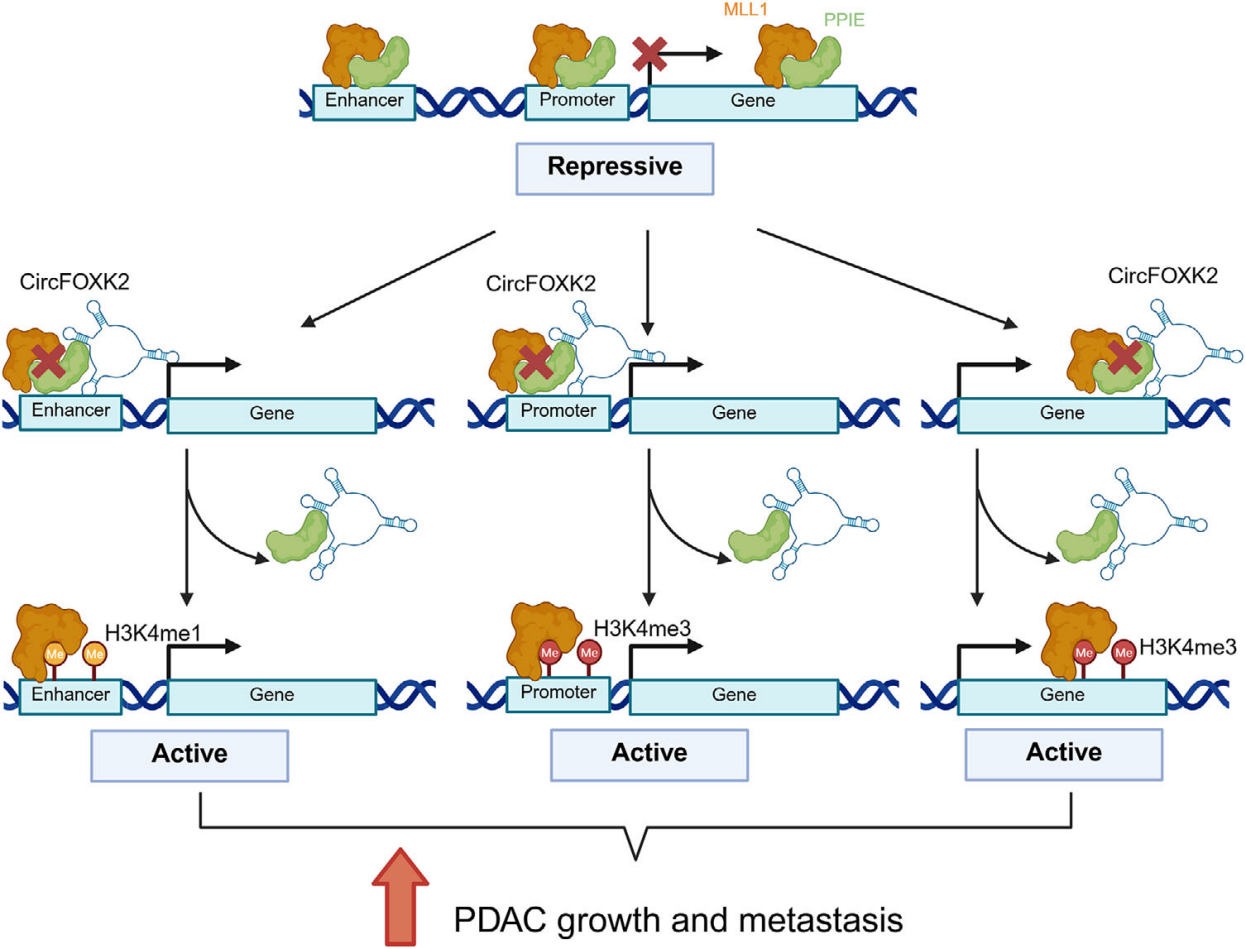
Schematic diagram showing the role of circFOXK2 in promoting H3K4me. ① PPIE interacts with MLL1 to inhibit H3K4me, resulting in gene repression. ② CircFOXK2 is frequently upregulated in many cancers, including PDAC. Through complementary base‐pairing, circFOXK2 recognizes the downstream target genes. Then, circFOXK2 with the AU‐rich motif binds to the RRM domain of PPIE and elicits it from MLL1. This removes the PPIE inhibitory effect on MLL1. ③ CircFOXK2 activates MLL1 at different genomic locations by sequence‐specific circRNA‐DNA interaction. CircFOXK2 promotes H3K4me1 at the enhancer and H3K4me3 at the promoter for gene activation. At the intragenic region, circFOXK2 facilitates transcription elongation by H3K4me3. The circRNA‐DNA interaction contributes to the oncogenic roles of circFOXK2 in promoting PDAC growth and metastasis. Created in BioRender.com.

## Author Contributions

CHW performed most of the experiments and drafted the manuscript. JJPP and SYL assisted in obtaining data. KFT and KSHC provided some research materials. YC was the PI of the grant, oversaw the whole process, and revised the manuscript.

## Funding

The work described in this paper was supported by grants from the National Natural Science Foundation of China (NSFC) (32270618) to YC; NSFC 82302905 to CHW; Strategic Seed Funding for Collaborative Research Scheme (SSFCR2324/8015) from CUHK; Research Matching Grant Scheme by UGC, the General Research Fund, Research Grants Council of Hong Kong Special Administrative Region, China (14120618 and 14120419); SBS Seed Fund for Collaborative Research (SFCR) (2022‐23) to YC and Research Impact Fund (R4007‐23).

## Ethics Statement

The study was carried out with the approval of the Joint CUHK‐NTEC Clinical Research Ethics Committee (Ref. No.: 2020.042).

## Patient Consent Statement

Written informed consent forms were signed by the patients.

## Conflicts of Interest

The authors declare no conflicts of interest.

## Supporting information




**Supporting File**: advs77107‐sup‐0001‐SuppMat.docx.

## Data Availability

The data that support the findings of this study are openly available in ProteomeXchange at http://www.ebi.ac.uk/pride/archive/projects/PXD015048, reference number PXD015048.
