# Supplementary material for: Genome‐Wide and Multi‐Omics Analysis Identified DNA‐Interacting Circular RNA circFOXK2 as MLL1 Regulator for Histone H3K4 Methylation in Pancreatic Ductal Adenocarcinoma
Source: Adv Sci (Weinh). 2026 Oct 29:e77107. Online ahead of print. doi: 10.1002/advs.77107 (PMC13717413; doi:10.1002/advs.77107)
Supplement: Supplementary file 1 [file ADVS-9999-e77107-s001.docx]

**Supplementary Figures**

**Figure S1 circFOKX2 binds to the target genes' DNA in PDAC cells.** (A) CircFOXK2 was knocked down by siRNA targeting the unique back-splicing junction. (B) ChIRP assay confirmed that circFOKX2 knockdown decreased its binding to the target genes' DNA in PDAC cells. Data represent mean ± SD from at least three independent experiments (*p < 0.05; **p < 0.01; ***p < 0.001).

**Figure S2 Full images of the immunoblotting showing the TRIO, LAMA4, BCAS1, TMSB15B, CYP26A1, TCP11, and GAPDH.**

**Figure S3 TRIO, LAMA4, TMSB15B, BCAS1, CYP26A1, and TCP11 were upregulated in PDAC.** (A) Analysis of the TCGA PAAD and the GTEx pancreatic dataset revealed the upregulated TRIO, LAMA4, TMSB15B, BCAS1, CYP26A1, and TCP11 in pancreatic cancer (n=350). (B) IHC analysis of PDAC tumors (our in-house cohort) consistently revealed the upregulated TRIO, LAMA4, TMSB15B, BCAS1, CYP26A1, and TCP11, as compared with adjacent non-tumor tissues. (C) Upregulated TRIO and LAMA4 were linked to poor disease-free survival. Survival analysis was performed by GEPIA2 using TCGA PAAD dataset. Data represent mean ± SD (***p < 0.001).

**Figure S4 TRIO promoted PDAC cell growth, migration, and invasion.** (A) TRIO was knocked down by siRNAs in PDAC cells. (B) TRIO knockdown inhibited PDAC cell growth, (C) clonogenic ability, (D) cell migration, and (E) cell invasion. Data represent mean ± SD from at least three independent experiments (**p < 0.01; ***p < 0.001).

**Figure S5 LAMA4 promoted PDAC cell growth, migration, and invasion.** (A) LAMA4 was knocked down by siRNAs in PDAC cells. (B) LAMA4 knockdown inhibited PDAC cell growth, (C) clonogenic ability, (D) cell migration, and (E) cell invasion. Data represent mean ± SD from at least three independent experiments (*p < 0.05; **p < 0.01; ***p < 0.001).

**Figure S6 TRIO knockdown rescued the oncogenic effects of circFOXK2.** CircFOXK2 overexpression in HPDE cells promoted clonogenic ability, which was rescued by TRIO knockdown. Data represent mean ± SD from at least three independent experiments.

**Figure S7 MYC regulates circFOXK2 expression in PDAC.** (A) Identification of the transcription factors binding to the promoter of circFOXK2 using the ENCODE and ChEA datasets. (B) ChIP-seq analysis using the ENCODE datasets confirmed the binding of MYC and FOS to the promoter of circFOXK2 in cancer cells. (C) PANC-1 snATAC-seq data analysis revealed the positive correlation between circFOXK2 and MYC gene activities.

**Figure S8 CircFOXK2-PPIE interaction removed the inhibitory effect on MLL1.** (A) CircFOXK2 knockdown in PANC-1 cells reduced its binding to PPIE. (B) ChIP-seq revealed the increased H3K4me3 level in PDAC, compared to the normal pancreas. (C) PPIE-binding sites on circFOXK2 were mutated. (D) The mutation reduced circFOXK2 binding to PPIE by RIP assay. (E) Full images of the immunoblotting showing the PPIE from the MLL1 Co-IP assay. (F) Co-IP analysis revealed that circFOXK2 overexpression inhibited MLL1 interaction with PPIE, which was restored by mutating the PPIE-binding sites. Data represent mean ± SD from at least three independent experiments (*p < 0.05; **p < 0.01; ***p < 0.001).

**Figure S9 Full images of the immunoblotting showing the PPIE, GAPDH, H3K4me1, and total H3.**

**Figure S10 CircFOXK2 promotes PDAC cell growth through the PPIE-MLL1 pathway.** (A, B) CircFOXK2 overexpression promoted HPDE cell growth, which was restored by either (A) mutating PPIE-binding sites or (B) MLL1 knockdown. Data represent mean ± SD from at least three independent experiments (***p < 0.001).

**Figure S11 Heat map and read density plot showing the H3K4me3 level, DNase-seq signals, and RNA PolII binding along the genes.** A positive correlation of read signals between H3K4me3 level, DNase-seq signals, and RNA PolII binding was observed in PDAC.

**Figure S12 MLL1 promoted TRIO, LAMA4, TMSB15B, BCAS1, CYP26A1, and TCP11 expression in PDAC.** (A, B) MLL1 knockdown in PDAC cells inhibited TRIO, LAMA4, TMSB15B, BCAS1, CYP26A1, and TCP11 expression at (A) RNA level and (B) protein level. (C) MLL1 knockdown inhibited H3K4me3, RNA PolII, and RNA PolII S2 levels on *TRIO* and *LAMA4* in PANC-1 cells. (D, E) MLL1 knockdown inhibited (D) chromatin accessibility and (E) transcription elongation on *TRIO* and *LAMA4* in PANC-1 cells. (F) MLL1 knockdown inhibited H3K4me3 levels on *TMSB15B* and *BCAS1* in PANC-1 cells. (G) MLL1 knockdown inhibited H3K4me1 levels on *CYP26A1* and *TCP11* in PANC-1 cells. (H) Positive correlation between MLL1, TRIO, LAMA4, TMSB15B, BCAS1, CYP26A1, and TCP11 expressions were observed in pancreatic cancer. Transcriptomic analysis of the pancreatic tumors was performed by GEPIA2 using TCGA PAAD dataset (n=178). Data represent mean ± SD from at least three independent experiments (*p < 0.05; **p < 0.01; ***p < 0.001).

**Figure S13 Sanger sequencing of the ChIP-PCR product targeting TRIO, LAMA4, BCAS1, TMSB15B, CYP26A1, and TCP11.**

**Figure S14 PPIE inhibited TRIO, LAMA4, TMSB15B, BCAS1, CYP26A1, and TCP11 expression in PDAC.** (A, B) PPIE knockdown in PDAC cells upregulated TRIO, LAMA4, TMSB15B, BCAS1, CYP26A1, and TCP11 expression at (A) RNA level and (B) protein level. (C) PPIE knockdown in PANC-1 cells promoted MLL1 binding and H3K4me3, RNA PolII, and RNA PolII S2 levels on *TRIO* and *LAMA4*. (D) PPIE knockdown in PANC-1 cells promoted chromatin accessibility on *TRIO* and *LAMA4*. (E) PPIE knockdown in PANC-1 cells increased MLL1 binding and H3K4me3 levels on *TMSB15B* and *BCAS1*. (F) PPIE knockdown in PANC-1 cells upregulated MLL1 binding and H3K4me1 levels on *CYP26A1* and *TCP11*. (G) ChIP analysis revealed that circFOXK2 overexpression in HPDE cells reduced PPIE binding on *TRIO* and promoted MLL1 binding, H3K4me3 level, and RNA PolII occupancy. This could be restored by mutating PPIE-binding sites on circFOXK2. (H) CircFOXK2 overexpression in HPDE cells promoted chromatin accessibility on *TRIO*, which could be inhibited by mutating PPIE-binding sites on circFOXK2. Data represent mean ± SD from at least three independent experiments (*p < 0.05; **p < 0.01; ***p < 0.001).

**Table S1. siRNA sequences for gene knockdown**

|  | Sense | Antisense |
| --- | --- | --- |
| si-circFOXK2 | GAAGGUGCACAUUCAGGUUTT | AACCUGAAUGUGCACCUUCTT |
| siMLL1 | CGAUCAAAUGCCCGCCUAATT | UUAGGCGGGCAUUUGAUCGTT |
| siPPIE-1 | AGCUAUCGACAACAUGAAUTT | AUUCAUGUUGUCGAUAGCUTT |
| siPPIE-2 | GAGACAUCACAGAUAUUCATT | UGAAUAUCUGUGAUGUCUCTT |
| siTRIO-1 | GUUCUCUAUGCUGGAAGCCTT | GGCUUCCAGCAUAGAGAACTT |
| siTRIO-2 | GUUUGAAUGAGCUCAGCCATT | UGGCUGAGCUCAUUCAAACTT |
| siLAMA4 | CAGGGAUUUAUGCAGAAAUTT | AUUUCUGCAUAAAUCCCUGTT |

**Table S2. Oligos used in this study**

| circFOXK2-F | CGGGGTCTTCAGGGTACAAGG |
| --- | --- |
| circFOXK2-R | GATGTTTGTGCTCGGGAACCT |
| MLL1-F | GTGCTTTGTGGTCAGCGGAAGT |
| MLL1-R | TGTGAGACAGCAACCCACGGTG |
| PPIE-F | CCTCGGGAAAACTCTGACCT |
| PPIE-R | ACTCAGATGGGGCTCAACTC |
| TRIO-F | TGATGGCTGCCTGGAATACA |
| TRIO-R | CTGTCCCTCCAAATCCAGGT |
| LAMA4-F | TTCGAACACCAGCTGACAAC |
| LAMA4-R | AGGTAACCATTGCGCATTTC |
| TMSB15B-F | CAGCAGGAGAAAGAGTGTGT |
| TMSB15B-R | ACCCCTGACCTCTTAACCAG |
| BCAS1-F | AGGAAGGCACCAAGGAGAAA |
| BCAS1-R | GCAGCATCTCCTTCGTTGAG |
| TCP11-F | GACAGCTCCAGAACATTGCC |
| TCP11-R | AACTTTTGGCCCAGTTCTGC |
| CYP26A1-F | CCAGAAAGTGCGAGAAGAGC |
| CYP26A1-R | AGTCTTCAGAGCAACCCGAA |
| TRIO-C-F1 | ACTGTGAGGATGGATGGCTC |
| TRIO-C-R1 | CCAGCAATCATCTCGCCATC |
| TRIO-C-F2 | CTGGGTCTGTGTGCAAATCATT |
| TRIO-C-R2 | GGTTGCCAGAGTGACAGTTAAT |
| TRIO-C-F3 | TTTCCTTTGCTGTCATTCCGTT |
| TRIO-C-R3 | CTTTAACACACAAGCACCAGGA |
| LAMA4-C-F1 | AGGCTTCTCAGTCCATAGGG |
| LAMA4-C-R1 | GCAGATGAATTAGGCAGGCA |
| LAMA4-C-F2 | GACAGGGCACACACGTTAAG |
| LAMA4-C-R2 | TGAGGCTTCGGAATGTGTCT |
| LAMA4-C-F3 | TCCTCTCCGTGTGCAGTATC |
| LAMA4-C-R3 | ATCCGTCTGCCTTTTGATGC |
| BCAS1-C-F1 | ACTCTTGGGGTGTCAGTGC |
| BCAS1-C-R1 | AGAATGCCAGTGAGTTCATGAC |
| BCAS1-C-F2 | GCACAGGTATTTACGGGCAG |
| BCAS1-C-R2 | TTGCGATTAGAATGCGGGTG |
| BCAS1-C-F3 | TAGGGCTGCTTGGTGATTGA |
| BCAS1-C-R3 | CCAGAGATGGTTGCCTTCCT |
| TMSB15B-C-F1 | TGACTTTTCTTGATGACTTCCCT |
| TMSB15B-C-R1 | GTGAGAAATAAAATGACAGGCCC |
| TMSB15B-C-F2 | GGAGATGCAAAAGGGGTTCA |
| TMSB15B-C-R2 | CTGGTTGCGCTGAGAAAGG |
| TMSB15B-C-F3 | TTCTTTACCTGCGCTCCACT |
| TMSB15B-C-R3 | CGACTCGGCTACCTCTCTG |
| CYP26A1-C-F1 | GAGTCAAACGGAAGGCAAGG |
| CYP26A1-C-R1 | ACCCACGCTCACATATTCCA |
| CYP26A1-C-F2 | CCCTCTGCTTTGTCACCCTA |
| CYP26A1-C-R2 | GCTGGCCAACACATCTGAAA |
| TCP11-C-F1 | TGATTTGCCTAGACTATCCTCGT |
| TCP11-C-R1 | TACCCAGCACTTTCCAACCA |
| TCP11-C-F2 | TAGCACACATCTCTGGGACC |
| TCP11-C-R2 | TCATGGGGCTGTGCTAAAGA |
| TCP11-C-F3 | CAGTTTCCACAGAACCGCAT |
| TCP11-C-R3 | AGCTCAACTCTGTCCTGCAA |
| GAPDH-F | TGCCTCCTGCACCACCAACT |
| GAPDH-R | CCCGTTCAGCTCAGGGATGA |

**Table S3. Genomic location of the circFOXK2-interacting DNA identified by ChIRP-seq with the top 20 binding score**

| Chr | Start | End | annotation | Gene | logFC | P-value | Adj. p-value |
| --- | --- | --- | --- | --- | --- | --- | --- |
| chr11 | 1022723 | 1023172 | Intron | MUC6 | 15.05244 | 1.96E-07 | 4.7E-05 |
| chr5 | 1.76E+08 | 1.76E+08 | Distal  Intergenic | UNC5A | 14.58103 | 4.44E-07 | 4.7E-05 |
| chr5 | 14326170 | 14326383 | Intron | TRIO | 14.45479 | 5.53E-07 | 4.7E-05 |
| chr10 | 1.13E+08 | 1.13E+08 | Distal  Intergenic | ADRA2A | 14.45053 | 5.56E-07 | 4.7E-05 |
| chr3 | 10501113 | 10501453 | Intron | ATP2B2 | 14.42034 | 5.87E-07 | 4.7E-05 |
| chr1 | 2.11E+08 | 2.11E+08 | Distal  Intergenic | KCNH1 | 14.1477 | 9.41E-07 | 4.7E-05 |
| chr5 | 1.37E+08 | 1.37E+08 | Intron | SPOCK1 | 14.12113 | 9.86E-07 | 4.7E-05 |
| chr3 | 12779885 | 12780220 | Exon | TMEM40 | 13.90766 | 1.43E-06 | 4.7E-05 |
| chr11 | 38780565 | 38780748 | Distal  Intergenic | LRRC4C | 13.83759 | 1.61E-06 | 4.7E-05 |
| chr20 | 36876082 | 36876356 | Intron | KIAA1755 | 13.82447 | 1.65E-06 | 4.7E-05 |
| chr5 | 1.55E+08 | 1.55E+08 | Distal  Intergenic | KIF4B | 13.80458 | 1.71E-06 | 4.7E-05 |
| chr3 | 1.35E+08 | 1.35E+08 | Distal  Intergenic | EPHB1 | 13.79788 | 1.73E-06 | 4.7E-05 |
| chrX | 71603104 | 71603324 | Intron | CITED1 | 13.79116 | 1.75E-06 | 4.7E-05 |
| chr3 | 62274663 | 62274993 | Intron | PTPRG-AS1 | 13.75705 | 1.85E-06 | 4.7E-05 |
| chr4 | 67354881 | 67355064 | Distal  Intergenic | EPHA5-AS1 | 13.74318 | 1.9E-06 | 4.7E-05 |
| chr1 | 1.82E+08 | 1.82E+08 | Exon | CACNA1E | 13.69355 | 2.07E-06 | 4.7E-05 |
| chr8 | 41075784 | 41075986 | Distal  Intergenic | SFRP1 | 13.69355 | 2.07E-06 | 4.7E-05 |
| chr4 | 1.65E+08 | 1.65E+08 | Intron | ANP32C | 13.68632 | 2.1E-06 | 4.7E-05 |
| chr4 | 67398908 | 67399091 | Distal  Intergenic | EPHA5-AS1 | 13.68632 | 2.1E-06 | 4.7E-05 |
